# Adeno-associated virus gene delivery targeting corneal-projecting trigeminal ganglion neurons

**DOI:** 10.3389/fncel.2026.1925806

**Published:** 2026-10-22

**Authors:** Samuel S. Reynolds, Md Zeeshan Rasheed, Ken Niyonkuru, Sachin Parikh, Kyle Whyland, Brooklynn M. Merrill, William Perez, Sreya Mitra, Tomasz Zal, Hongmin Yun, Brian Davis, Anthony J. St. Leger, Anna Matynia, Ian D. Meng

**Affiliations:** 1 Center for Excellence in the Neurosciences, University of New England, Biddeford, ME, United States; 2 Graduate School of Biomedical Science and Engineering, University of Maine, Orono, ME, United States; 3 College of Optometry, University of Houston, Houston, TX, United States; 4 Department of Ophthalmology, Jules Stein Eye Institute, University of California, Los Angeles, Los Angeles, CA, United States; 5 Department of Ophthalmology, University of Pittsburgh, Pittsburgh, PA, United States; 6 Department of Neurobiology, University of Pittsburgh, Pittsburgh, PA, United States; 7 Department of Immunology, University of Pittsburgh, Pittsburgh, PA, United States

**Keywords:** adeno-associated virus, circuit mapping, circuit tracing, corneal innervation, gene delivery, PNS—peripheral nervous system, TRIGEMINAL ganglion neuron

## Abstract

Corneal innervation is essential for ocular surface homeostasis and protection, yet methods for selectively labeling and manipulating cornea-projecting neurons remain limited. Conventional retrograde tracers can identify cornea-projecting cell bodies in the trigeminal ganglion (TG), but they do not enable the expression of genetically encoded effector proteins, sensors, or recombinases. We describe and validate intrastromal adeno-associated virus (AAV) delivery as an approach for labeling cornea-projecting TG neurons in mice. A 1–2-μL viral injection was delivered into the corneal stroma and labeling was assessed 2–12 weeks later in cleared whole TG, TG sections, corneal whole mounts, and thick brainstem sections. Multiple AAV vectors, including tdTomato-, eGFP-, or mCherry-expressing vectors and Cre-expressing vectors in Ai14 tdTomato reporter mice, produced consistent neuronal labeling concentrated in the dorsomedial ophthalmic division of the TG. Native tdTomato fluorescence was readily detected, and eGFP labeling could be enhanced using immunolabeling with fluorophore-enhancing antibodies, increasing detection of labeled somata and axonal fibers. AAV-mediated labeling in the TG substantially overlapped with WGA and Fast Blue tracing from the cornea, although AAV labeled fewer neurons overall. Cre-expressing AAV vectors activated reporter expression in Ai14 mice, demonstrating compatibility with transgenic reporter strategies. Labeled axons were visualized at both peripheral and central projection sites. At the ocular surface, reporter expression was correlated to the extent of neuronal transduction. Central projections were observed in subnucleus principalis, the interpolaris/caudalis transition zone, and caudal subnucleus caudalis extending into upper cervical spinal cord. AAV transduction resulted in no gross immune activation. These findings indicate that intrastromal AAV delivery provides a versatile approach for anatomical tracing and genetic access to cornea-projecting neurons, enabling expression of genetically encoded tools for optogenetic and chemogenetic control, activity monitoring, and targeted genetic manipulation.

## Introduction

Sensory innervation of the cornea is central to ocular surface homeostasis, protection, and post-injury repair (Muller et al., 2003; Shaheen et al., 2014; Yang et al., 2018; Labetoulle et al., 2019). Corneal afferents coordinate protective reflexes and behaviors, including tear secretion and blinking, in response to mechanical, thermal, chemical, and osmotic stimuli (Acosta et al., 2004; Belmonte et al., 2017; Meng and Kurose, 2013; Parra et al., 2014, 2010). Because the vast majority of these corneal neurons are nociceptive, they are exquisitely tuned to detect these sensory inputs as potential pain or threat. These nerves also interact closely with the epithelial and stromal microenvironments, regulating epithelial maintenance, inflammation, and wound healing (Feinberg et al., 2023; Liu et al., 2022; Jones and Marfurt, 1998; Okada et al., 2019). As a result, altered corneal sensory signaling can contribute to ocular surface disorders such as neuropathic corneal pain, dry eye disease, and neurotrophic keratopathy (Sacchetti and Lambiase, 2014; Craig et al., 2017; Goyal and Hamrah, 2016; Galor et al., 2016). Methods providing selective access to cornea-projecting neurons would offer powerful tools for studying the role of corneal afferents in health and disease while supporting the development of targeted strategies to treat ocular surface disorders.

The cornea, innervated by A-delta and C fiber primary afferent neurons, is among the most densely innervated tissues in the body (Muller et al., 2003; MacIver and Tanelian, 1993; Zander and Weddell, 1951; Rozsa and Beuerman, 1982; Muller et al., 1996, 1997). Conventional retrograde and transganglionic tracers, including WGA, HRP-WGA, Fluoro-Gold, Fast Blue (FB), and cholera toxin B, have provided much of the current understanding of cornea-projecting neurons (Felipe et al., 1999; Marfurt et al., 1989; Marfurt and Echtenkamp, 1988; Marfurt and Del Toro, 1987; LaVail et al., 1993; Nakamura et al., 2007; Hegarty et al., 2010; Aicher et al., 2013). The majority of these neurons are sensory, with cell bodies located in the ophthalmic division (V1) of the trigeminal ganglia (TG). Sympathetic fibers with cell bodies in the superior cervical ganglion and parasympathetic fibers with cell bodies in the ciliary and accessory ciliary ganglia make a relatively minor, species-specific contribution (Labetoulle et al., 2019; Jones and Marfurt, 1998; Marfurt et al., 1989; Marfurt and Ellis, 1993). A substantial portion of cornea-projecting TG neurons are peptidergic, expressing calcitonin gene-related peptide (CGRP) and/or substance P (Jones and Marfurt, 1998; Felipe et al., 1999; LaVail et al., 1993; Nakamura et al., 2007; Hegarty et al., 2010; He and Bazan, 2016; Murata and Masuko, 2006; Alamri et al., 2015; Sullivan et al., 2022; Lehtosalo, 1984). Their central processes project into the trigeminal brainstem complex, where corneal input has been described in two primary regions: the interpolaris/caudalis transition zone and caudal subnucleus caudalis which extends into the upper cervical spinal cord (Marfurt and Echtenkamp, 1988; Marfurt and Del Toro, 1987; Hegarty et al., 2010; Aicher et al., 2013; Marfurt, 1981; Hirata et al., 2003; Meng et al., 1997; Panneton and Burton, 1981; Panneton et al., 2010; Hirata et al., 2004). Additional projections have been reported in trigeminal subnucleus principalis, although this may be species dependent (Marfurt and Echtenkamp, 1988; Panneton and Burton, 1981; Panneton et al., 2010). These approaches have provided important anatomical insight into corneal sensory pathways, but they do not provide genetic access to cornea-projecting neurons, or enable expression of genetically encoded reporters, recombinases, sensors, or effector proteins.

Recombinant adeno-associated viruses (AAVs) offer a potential strategy to access cornea-projecting neurons. AAVs are replication-deficient viral vectors that can deliver transgene cassettes to target cell populations (Samulski and Muzyczka, 2014; Wang et al., 2019; Naso et al., 2017). Different AAV serotypes and capsids vary in the cell types they preferentially transduce, the efficiency with which they drive transgene expression, and their capacity for axonal uptake and transport, allowing vector selection to be tailored to specific tissues or applications (Wu S. et al., 2006; Wu Z. et al., 2006; Aschauer et al., 2013; Murlidharan et al., 2014; Salegio et al., 2013; Tervo et al., 2016). AAVs have been widely used to express fluorescent reporters, recombinases, and tools for monitoring or manipulating defined populations of neurons, including optogenetic actuators, chemogenetic receptors, and calcium indicators (Armbruster et al., 2007; Boyden et al., 2005; Roth, 2016). Specific to the cornea, intrastromal AAV delivery has been used for viral transduction of stromal cells and keratocytes and in preclinical studies as a therapeutic approach for corneal fibrosis, neovascularization, and corneal clouding caused by the inherited stromal disease mucopolysaccharidosis type I (Gupta et al., 2017; Vance et al., 2016; Hirsch et al., 2017; Miyadera et al., 2020; Carlson et al., 2004; Hippert et al., 2012; Matthaei et al., 2012; Mohan et al., 2011). More recent studies have shown that intrastromal AAV-mediated nerve growth factor delivery can transduce corneal nerves and promote nerve regeneration in neurotrophic keratopathy and diabetic keratopathy mouse models (Cong et al., 2024, 2025).

In this study, we evaluated intrastromal AAV delivery as a strategy for reproducible genetic access to cornea-projecting TG neurons. Using multiple AAV vectors across independent laboratories, we assessed viral labeling in the cornea, TG, and trigeminal brainstem complex and benchmarked AAV-mediated transduction against conventional retrograde tracers. We also tested Cre-expressing AAV vectors in reporter mice to determine whether intrastromal delivery could support transgenic recombination-based approaches. Together, these experiments establish intrastromal AAV delivery as a reliable and versatile platform for genetically labeling cornea-projecting TG neurons and mapping their peripheral and central projections, with potential applications for future manipulation and monitoring of corneal sensory pathways.

## Methods

### Animals

Male and female Ai14 (#007914) (Madisen et al., 2010), C57BL/6J (#000664), Scn10a^Cre^ (#036564)(Nassar et al., 2004), Rosa26-DTA176 (#010527) (Wu S. et al., 2006) (The Jackson Laboratory, Bar Harbor, ME, USA), the double mutant Scn10a^Cre^; Rosa26-DTA176 resulting in ablation of the NaV1.8-expressing neurons and Scn10a^Cre − tdTom^ mice (Shields et al., 2012) (a generous gift of Dr. Dib-Hajj), 5 weeks to 6 months of age, were housed on a controlled 12-h light/dark cycle with *ad libitum* access to food and water. Animals were treated in accordance with National Institutes of Health guidelines for the care and use of laboratory animals and the ARVO Statement for the Use of Animals in Ophthalmic and Vision Research. All procedures were approved by the Institutional Animal Care and Use Committee at the University of New England, the University of California at Los Angeles and the University of Houston.

### Adeno-associated viruses

Recombinant adeno-associated viral vectors (AAVs) were used for fluorescent labeling and Cre delivery. AAV9, AAV-PHP.S, and AAVrg vectors carrying CAG-tdTomato were obtained from Addgene (cat. #59462-AAV9, #59462-PHP.S, and #59462-AAVrg) and used to drive tdTomato expression. AAV1, AAV2-7m8, AAV6 and AAV9 carrying CMV- or CAG-driven eGFP or mCherry were obtained from Vector Biolabs (cat. #7086-AAV1, #7600-AAV2-7m8, #7019-AAV6-CMV-eGFP, #7074-AAV6-CAG-eGFP, #VB1334-AAV6-CAG-mCherry, #custom-AAV6-CMV-mCherry) and AAV8-CAG-eGFP was obtained from PackGene (#custom) to drive reporter expression. In addition, AAVrg-hSyn-Cre-P2A-dTomato (Addgene cat. #107738-AAVrg) and AAVrg-CAG-Cre-P2A-mNeptune (COVF Viral Vector Core; RRID:SCR_016477) were used to drive Cre expression in Ai14 tdTomato reporter mice. All viruses had titers at or above 1 × 10^13^ vg/mL. AAVs were aliquoted, stored at −80 °C, and used undiluted unless otherwise specified.

To compare AAV-mediated labeling with wheat germ agglutinin (WGA) tracing, WGA conjugated to Alexa 488 (Biotium, #29076-1) or Alexa 647 (Thermo Fisher, #W32466) was used. WGA tracers were diluted 1:1 according to the manufacturers' recommendations and stored at −20 °C.

### Intrastromal injections

Intrastromal injections were performed in 5-week to 6-month-old mice under 2% isoflurane. Sterile normal saline was applied to the ocular surface to maintain hydration, and the eye was gently proptosed using forceps. Under a surgical microscope, a 30-gauge × 1/2-in needle was used to create a small opening in the center of the cornea (Carlson et al., 2004). A 5-μL glass Hamilton syringe fitted with a 33-gauge needle with a 30–45° beveled tip was inserted through the corneal opening into the stromal tissue. Alternatively, a 35-gauge beveled needle was used to directly inject into the stroma. A total volume of 1–2 μL was injected into the intrastromal space, and the ocular surface was flushed with sterile saline after injection. In a subset of animals, WGA was injected 4 weeks after AAVrg injection.

### Perfusions and tissue preparation

Mice were perfused with 0.01 M phosphate-buffered saline (PBS) followed by 4% paraformaldehyde (PFA) 3–22 weeks after AAV injection or 5–7 days after WGA injection. After perfusion, trigeminal ganglia (TG) designated for frozen sectioning were removed and post-fixed in 4% PFA for 20 min before transfer to 30% sucrose. Trigeminal ganglia designated for tissue clearing and whole-ganglion imaging were post-fixed in 4% PFA for at least 3 h before transfer to PBS containing 0.02% sodium azide. For evaluation of central projections, brains through the second cervical vertebra were removed, post-fixed in 4% PFA for 3 days, and stored in PBS containing 0.02% sodium azide before vibratome sectioning.

Corneas were excised from enucleated eyes under a dissecting microscope, post-fixed in 4% PFA for 20 min or 1 h and transferred to PBS containing 0.02% sodium azide. Three to five radial cuts were made with micro-scissors or a scalpel blade to allow corneas to lie flat on slides. Whole corneas were incubated with the nuclear fluorescent dye4′,6-diamidino-2-phenylindole (DAPI; 1:1,000) or Hoechst 33342 (1:10,000 Sigma, B2261) for 15 min, washed in PBS for 10 min, positioned posterior side down on glass slides, mounted with Fluoromount-G (SouthernBiotech), coverslipped, and sealed with clear nail polish.

### Immunofluorescence labeling and imaging of trigeminal ganglia sections

Trigeminal ganglia were embedded in Tissue-Tek optimal cutting temperature compound (Sakura Finetek, USA) and frozen in an isopentane bath on dry ice or by placing the mold directly onto dry ice. Serial 12-μm to 20-μm sections were cut on a cryostat and mounted on slides for immunofluorescence antibody staining. Slides were washed in PBS and blocked for 1 h in PBS containing 5% normal donkey serum (NDS) and 0.1% or 0.3% Triton X-100. Sections were incubated overnight at room temperature in rabbit anti-calcitonin gene-related peptide (CGRP) primary antibody (1:1,000; CB198, lot 341558), or at 4 °C with chicken anti-GFP primary antibody (1:1,000, Fisher, #A10262), chicken anti-mCherry primary antibody (1:1,000, Novus, #NBP2-25158) or rat anti-CD206 primary antibody (1:200, BioLegend, #141701) diluted in blocking solution. Slides were then washed three times for 10 min each in blocking solution and incubated for 2 h at room temperature with Alexa Fluor 647-conjugated donkey anti-rabbit secondary antibody (1:500; Jackson ImmunoResearch, #711-605-152, lot #172036), Alexa Fluor 488-conjugated goat anti-chicken (1:1,000; Fisher, #A11039) or Alexa Fluor 488-conjugated donkey anti-rat (1:1,000; Fisher, #A21208). Slides were washed three times for 10 min each in PBS-Tx (PBS containing 0.2% Triton X-100), incubated with DAPI or Hoechst 33342 (1:10,000, Sigma, B2261), and coverslipped before imaging. Sections were imaged with an Olympus VS200 slide scanner, Fluoview 1000 or Leica Thunder. Cornea-projecting neurons that were double-labeled for CGRP were manually counted in Fiji.

### Tissue clearing and immunofluorescence of whole trigeminal ganglia and thick brain sections

For tissue-clearing experiments, brains were embedded in 5% agarose and sectioned at 300 μm using a Leica VT1000S vibratome. Sections were washed in 1× PBS. Whole TG prepared for clearing were post-fixed in 4% PFA for 24 h. Brain sections and whole TG were incubated in SHIELD-OFF solution (50% SHIELD-EPOXY, 25% SHIELD Buffer, and 25% deionized water (LifeCanvas)) at 4 °C for 24 h. Samples were transferred to SHIELD-ON solution (7:1 SHIELD-ON:SHIELD-EPOXY; LifeCanvas) for 3 h at 4 °C and then for an additional 3 h at room temperature. Samples were then incubated in delipidation buffer (LifeCanvas) overnight at 37 °C, followed by three 15-min washes in 1x PBS.

For immunolabeling, samples were incubated for 24 h at 37 °C in blocking solution containing 5% NDS and 0.4% Triton X-100 in PBS. Brain sections and TG were incubated with rabbit anti-NeuN primary antibody (1:500; Cell Signaling, #36662) for 48 h at 37 °C. For TG samples in which corneas were injected with AAV expressing eGFP, chicken anti-GFP antibody (1:500; Aves Labs, #GFP-1020) was included. Samples were washed three times in PBS-Tx (PBS containing 0.2% Triton X-100) and fixed in 4% PFA for 1 h at room temperature. Samples were then washed five times for 15 min each in PBS-Tx and incubated for 24 h at 37 °C with Alexa Fluor 647-conjugated donkey anti-rabbit (1:500; Jackson ImmunoResearch, #711-605-152); TG samples in which corneas were injected with AAV expressing eGFP were additionally incubated with Alexa Flour 568-conjugated donkey anti-chicken (1:500; Jackson ImmunoResearch, #703-575-155). Samples were washed three times in PBS-Tx and fixed again in 4% PFA for 1 h. After post-fixation, samples were washed five times for 15 min each in PBS-Tx, incubated with DAPI for 2 h at 37 °C, and washed twice for 15 min each in 1× PBS before index matching.

Samples were incubated in 50% EasyIndex (LifeCanvas) diluted in deionized water for 3 h at 37 °C, followed by incubation in 100% EasyIndex at 37 °C until transparent, ~2.5 h. Sections were mounted in EasyIndex and coverslipped with #1 cover glass.

### Imaging and analysis of whole trigeminal ganglia

Light-sheet imaging was performed with a LifeCanvas Technologies SmartSPIM using 488-, 561-, and 640-nm laser excitation and Chroma ET525/50m, ET600/50m, and ET690/50m emission filters, respectively. Samples were imaged with either a 3.6× objective (1.8 μm/pixel) or a 9.0× objective (0.71 μm/pixel), with a 2-μm z-step. Samples were imaged in immersion oil with a refractive index of 1.52, matched to EasyIndex.

Complete sample images were acquired as 16-bit TIFF files for each channel and z-slice. Images were destriped and stitched using the SmartSPIM post-processing pipeline (LifeCanvas Technologies, USA) and compiled into Imaris file format using Imaris File Converter (Oxford Instruments, UK). Volumetric images of cleared whole TG were analyzed in Imaris to quantify cells labeled with tdTomato or other applicable markers. The Imaris Spots algorithm was used to identify soma-candidate features, and a machine-learning classifier was used to distinguish neuronal soma from non-soma features. The cell-counting algorithm was trained on representative images and applied to automate quantification of soma for each label type.

To determine colocalization between labels, identified soma in one channel were filtered by proximity to identified soma in the other channel. Soma labels were considered overlapping when the centroid-to-centroid distance was less than the average soma diameter. Neuronal soma size was quantified from light-sheet fluorescence microscopy z-stacks of cleared whole trigeminal ganglia. Each labeled neuron was followed through the z-axis, and the X-Y optical plane in which the soma exhibited its largest cross-sectional profile was selected, similar to approaches utilized in light-sheet images of cleared trigeminal in rat (Kuramoto et al., 2024). The perimeter of the soma was manually traced in this plane, and the enclosed area was converted to an equivalent circular diameter in micrometers, as described in previous methods utilized in macaque monkey trigeminal ganglia (Usman and Balaban, 2016).

### Imaging of brain sections

Images were acquired using a Leica Stellaris laser-scanning confocal microscope. Excitation was performed using 405- and 561-nm diode lasers. Laser intensity and gain were adjusted to avoid pixel saturation while minimizing photobleaching and noise (~5–10% intensity and 2.5–10% gain). Overview images preserving anatomical context of labeled projections were acquired with an HC PL FLUOTAR 10×/0.30 dry objective. Z-stacks were collected from the top to the bottom of each section at 10-μm intervals, with a pixel size of 3.03 × 3.03 μm. Higher-magnification images of labeled projections were acquired with an HC PL FLUOTAR L 20×/0.40 dry objective. Z-stacks were collected from the top to the bottom of each section at system-optimized 2.41-μm intervals, with a pixel size of 0.38 × 0.38 μm. All z-stacks were processed as maximum-intensity projections, and lookup tables were adjusted for clarity.

### Imaging of cornea whole-mounts

Whole-mounted corneas were fixed for 20 min in 4% PFA then incubated in blocking solution containing 5% NDS and 0.3% Triton X-100 with 0.1% Tween-20 in PBS (PBS-Tx-Tw) overnight at 4 °C. Corneas were incubated with rabbit anti-TuJ1 primary antibody (1:10,000; BioLegend, #802001) and chicken anti-GFP primary antibody (1:1,000, Fisher, #A10262) in blocking solution for 5 days at 4 °C. Samples were washed three times in PBS-Tx-Tw (PBS containing 0.2% Triton X-100) and incubated for 24 h at 4 °C with secondary antibodies: Alexa Fluor 594-conjugated donkey anti-rabbit (1:1,000; Fisher, #A21207) and Alexa Fluor 488-conjugated goat anti-chicken (1:1,000; Fisher, #A11039). Samples were washed three times in PBS with the inclusion of Hoechst 33342 (1:10,000, Sigma, B2261) in the first wash and mounted with relieving cuts in Fluoromount-G (refractive index 1.40, Fisher Scientific, #OB10001).

To determine the percentage of corneal nerve fibers expressing the viral reporter, single confocal images were captured using single frame of view scan centered on the whorl using z-stacks of 3 μm steps as experimentally determined to separate nerve compartments with consistent nerve densities. Detector gain and laser intensity were manually optimized for each acquisition. Fluoview software was used to acquire images and export oib files for processing in Fiji. A custom plugin (corneal_nerves) for FIJI/ImageJ (github.com/yuekanjiao/corneal-nerves) was used to project the stromal nerves, sub-basal plexus and the intraepithelial terminals as distinct layers and generate a percent of pixels containing signal. A mathematical cutoff based on fluctuation of the first derivative of the dropping rate of the histogram peak of pixel intensity was used as a threshold, which was manually adjusted to adjust for background if needed.

### Statistical analysis

Quantitative analyses were performed using GraphPad Prism v10.1 (GraphPad Software, Boston, MA, USA). Data are presented as group mean ± SEM. Comparisons between two independent groups were analyzed using unpaired, two-tailed Student's t-tests, followed by Welch's correction. Comparisons among more than two groups were analyzed using ordinary one-way ANOVA followed by Tukey's multiple-comparisons test. Multiplicity-adjusted *P* values were used for *post hoc* comparisons. Statistical significance was set at *P* < 0.05.

## Results

### Whole-tissue light-sheet imaging of cornea projecting neurons

To compare the ability of different AAVs to label cornea-projecting TG neurons, intrastromal injections were performed using AAVs expressing fluorescent reporters, including tdTomato or eGFP, as well as AAVs expressing Cre in Ai14 tdTomato reporter mice. Trigeminal ganglia were cleared and imaged by light-sheet microscopy to visualize labeled neurons throughout the whole ganglion. Across all five AAVs examined, intrastromal delivery produced consistent and robust labeling of neuronal cell bodies localized mainly in the dorsomedial aspect of the ophthalmic division (V1), corresponding to the expected region of cornea-projecting neurons (Figures 1–3).

**Figure 1:**
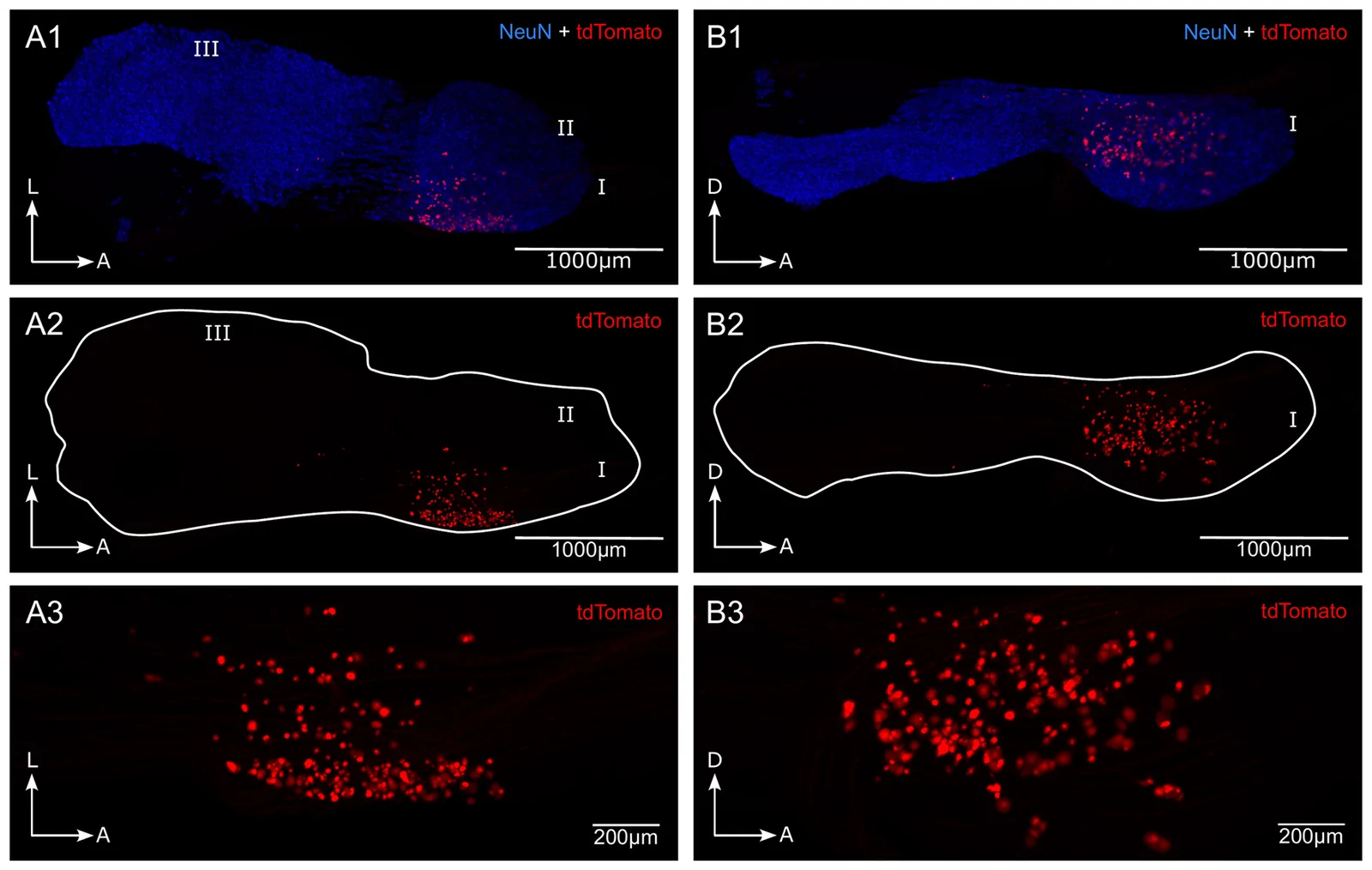
Imaris 3D rendering of LSFM image of cleared trigeminal ganglion after intrastromal corneal injection of Cre-expressing AAV2-retro virus under a hSyn promotor in Ai14; C57BL/6J mice. **(A1–3)** View from dorsal aspect, anterior end oriented toward right. **(B1–3)** View from medial aspect. **(A1, B1)** tdTomato-labeled cell soma (red) and NeuN channel (blue). **(A2, B2)** tdTomato only with outline indicating tissue boundaries. **(A3, B3)** Detail of corneal niche. Labeled neurons were largely restricted to dorsal branch I, anterior to branch III. A, Anterior; D, Dorsal; L, Lateral; I, II, III, trigeminal nerve branches.

For AAVs producing tdTomato signal either directly or through Cre-dependent recombination in Ai14 reporter mice, labeled neurons were readily detected using native tdTomato fluorescence, a known strong fluorophore, without additional immunofluorescence amplification (Figures 1, 3). In contrast, native eGFP fluorescence was enhanced by immunofluorescence using an anti-GFP antibody and a 568-conjugated secondary antibody. Consistent with the literature (Condro et al., 2016), antibody amplification increased the signal intensity, which was particularly noticeable in the improved labeling of axonal fibers (Figure 2). To examine the relationship between native eGFP fluorescence and the antibody-amplified signal, GFP-positive neurons were quantified using an Imaris machine learning-based automated cell detection workflow. The mean number of eGFP-positive neurons identified by native GFP fluorescence was significantly less than the number detected after GFP antibody amplification (74.3 ± 25.25 vs. 118.5 ± 29.5, *p* < 0.05, paired *t*-test). Among all neurons identified, 56% were labeled by both native GFP fluorescence and GFP immunoreactivity. An additional 40% were detected only after GFP antibody amplification, and 4% were detected only by native GFP fluorescence. The small fraction of native GFP-only neurons indicates broad antibody penetration throughout the cleared tissue sample, while the larger antibody-only population demonstrates the increased sensitivity produced by immunofluorescence amplification.

**Figure 2:**
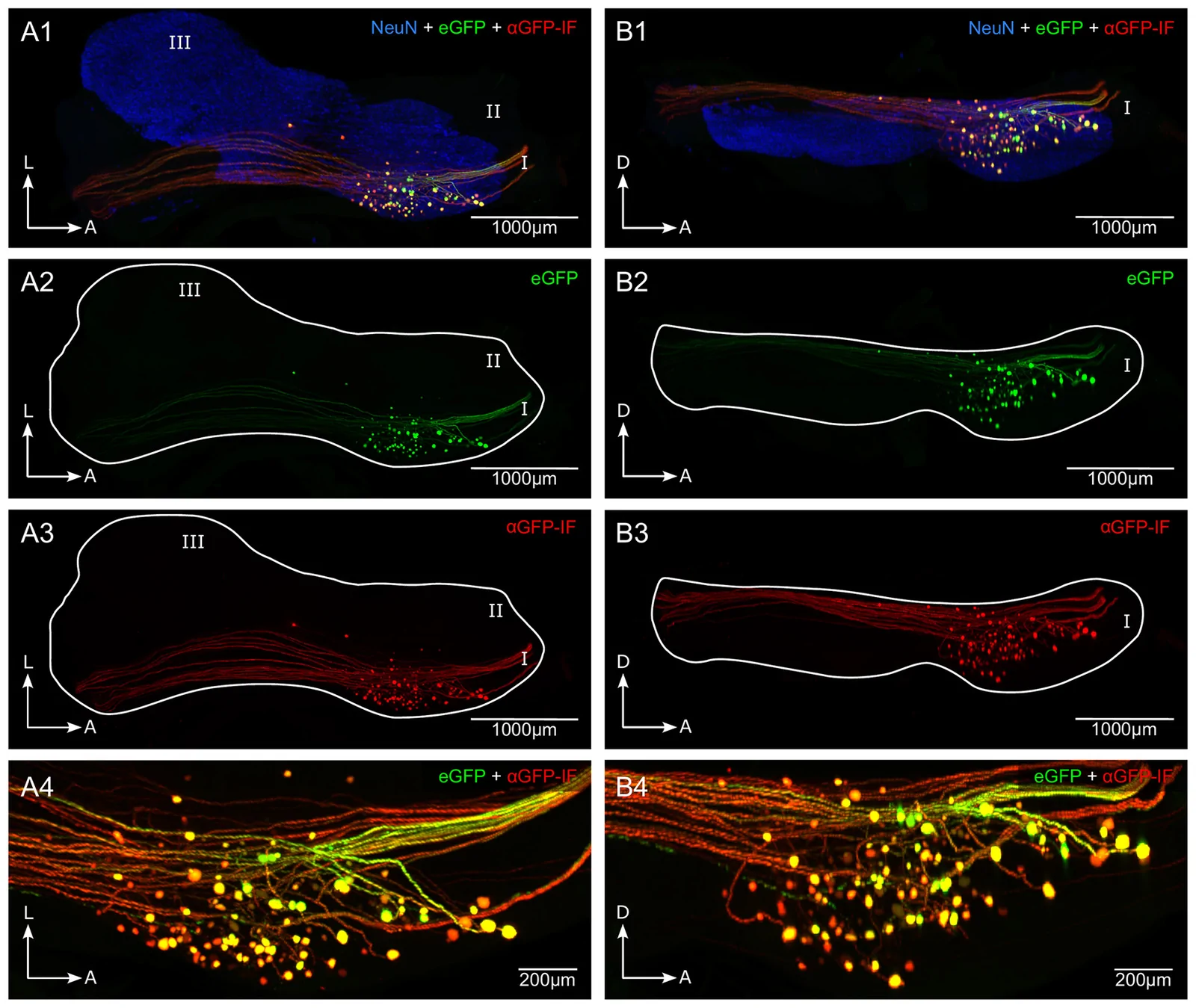
Imaris 3D rendering of LSFM image of cleared trigeminal ganglion after intrastromal corneal injection of eGFP-expressing AAV6 virus under a CMV promotor in C57BL/6J mice. Immunofluorescence (IF) staining was performed for GFP with secondary antibody in the 561 channel. **(A1–4)** View from dorsal aspect, anterior end oriented toward right. **(B1–4)** View from medial aspect. **(A1, B2)** eGFP (green) and αGFP-IF (red) labeled cell soma with NeuN (blue). **(A3, B3)** eGFP or αGFP-IF only with outlines of tissue. **(A4, B4)** Detail of eGFP and αGFP-IF co-labeling. IF for GFP improved overall signal intensity and consistency, increasing visibility of fibers and number of soma detectable by Imaris cell counting tools. A, Anterior; D, Dorsal; L, Lateral; I, II, III, trigeminal nerve branches.

**Figure 3:**
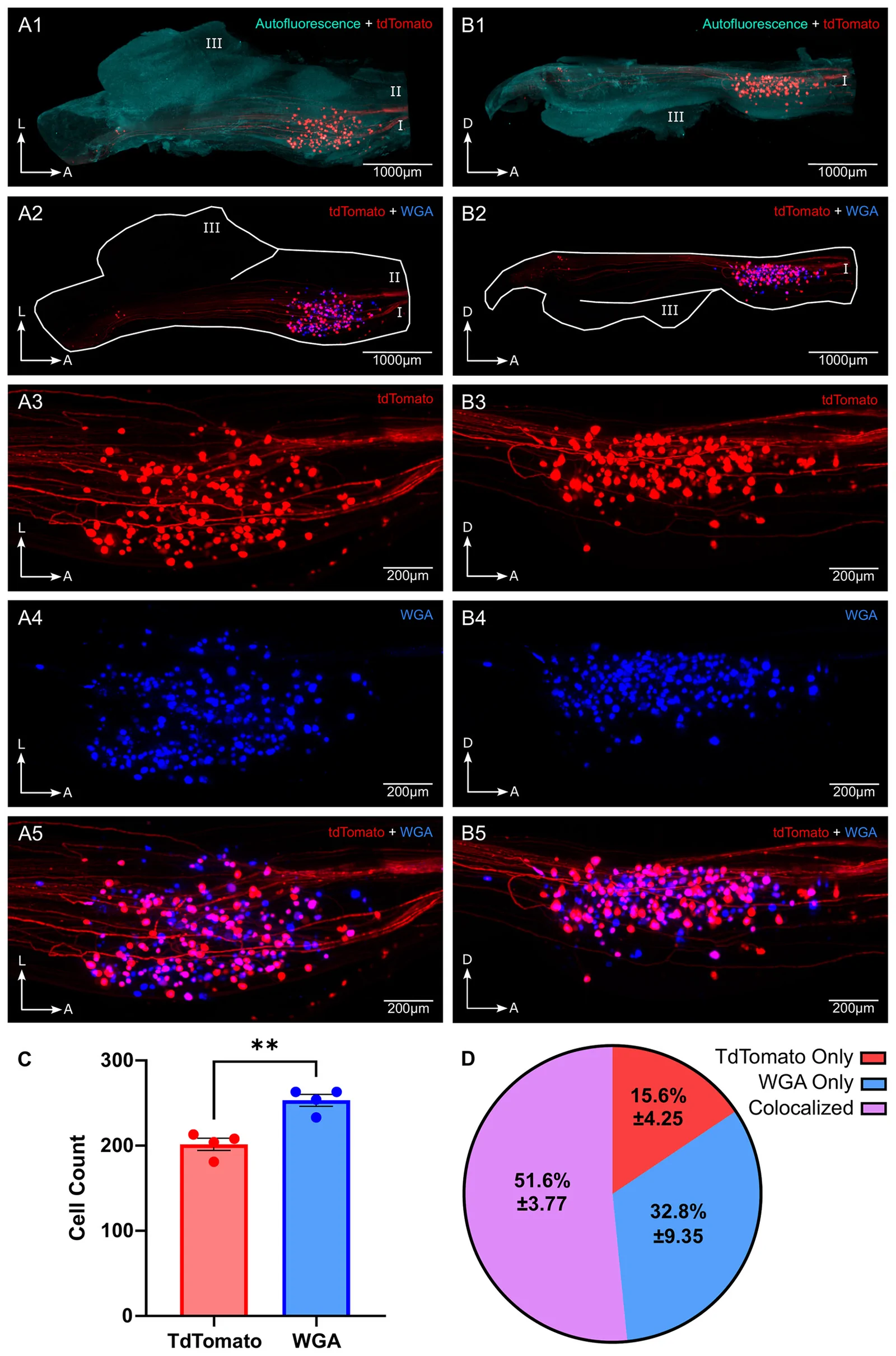
Cleared trigeminal ganglion after intrastromal corneal injection of tdTomato-expressing AAV2-retro virus under a CAG promotor and WGA tracer in C57BL/6J mice. **(A1–5)** View from dorsal aspect, anterior end oriented toward right. **(B1–5)** View from medial aspect. **(A1, B1)** tdTomato (red) and WGA (blue) labeled cell soma with autofluorescence (cyan). **(A2, B2)** tdTomato and WGA labeled cell soma with outline of tissue. **(A3–5, B3–5)** Details of tdTomato, WGA, and combined labels. Both tdTomato and WGA labeled cells were contained to the same anatomical region. A, Anterior; D, Dorsal; L, Lateral; I, II, III, trigeminal nerve branches. **(C)** Comparison of counts for tdTomato vs. WGA labeled cell soma using Imaris machine learning algorithm. ** *p* < 0.01, paired *t*-test. **(D)** Proportion of cell soma co-labeled by tdTomato and WGA vs. tdTomato only vs. WGA only.

### Quantification of the total number of labeled neurons after intrastromal injections of multiple AAVs was compared

AAV1-CMV-mCherry, AAV2-7m8-CAG-eGFP, AAV2-retro-CAG-tdTomato, AAV2-retro-CAG-Cre, AAV2-retro-hSyn-Cre, AAV6-CMV-eGFP, AAV6-CAG-eGFP, AAV6-CMV-mCherry, AAV6-CAG-mCherry, AAV8-CAG-eGFP, AAV9-CMV-eGFP, and AAV9-CAG-tdTomato were injected into the corneal stroma (Table 1). A selection of these AAVs representing different serotypes and promoters were quantitated for the total number of labeled neurons in cleared whole TGs: AAV2-retro-CAG-tdTomato, AAV2-retro-CAG-Cre, AAV2-retro-hSyn-Cre, and AAV6-CMV-eGFP were quantified, with AAV6-CMV-eGFP following immunofluorescence amplification (Figure 4). Although the mean number of labeled neurons varied numerically across viral constructs, with AAV6-CMV-eGFP showing the lowest average labeling, a one-way ANOVA revealed no significant difference between treatment groups (*p* > 0.05). Notably, the two Cre-expressing AAV2-retro vectors produced robust labeling in tdTomato reporter mice using either the CAG or hSyn promotor.

**Table 1:** Quantification of labeled neurons.

Virus	Tissue	*n*	Lab A	*n*	Lab B
AAV-PHP.S-CAG-tdTomato	Sections	6	71.50 ± 8.409		
AAV9- CAG-tdTomato	Sections	6	103.8 ± 5.510		
AAV2-retro-CAG-tdTomato^*^	Sections	6	106.8 ± 1.815		
AAV2-retro-CAG-tdTomato	Cleared	4	182.3 ± 14.14		
AAV2-retro-CAG-Cre	Cleared	4	219.8 ± 23.82		
AAV2-retro-hSyn-Cre	Cleared	4	197.3 ± 28.46		
AAV6-CMV-eGFP	Cleared	4	74.25 ± 25.15		
AAV6-CMV-eGFP^**^	Cleared/IF	4	118.5 ± 29.52		
AAV1-CMV-mCherry	Sections/IF			3	89.3 ± 38.8
AAV2-7m8-CAG-eGFP	Sections/IF			2	4.5 ± 4.5
AAV2-retro-CAG-tdTomato^*^	Sections			14	204.6 ± 20.8
AAV6-CAG-eGFP	Sections/IF			8	182.5 ± 40.3
AAV6-CMV-eGFP^**^	Sections/IF			16	92.3 ± 14.1
AAV6-CAG-mCherry	Sections/IF			8	81.3 ± 24.8
AAV6-CMV-mCherry	Sections/IF			16	18.0 ± 6.4
AAV8-CAG-eGFP	Sections/IF			4	36.7 ± 21.6
AAV9-CMV-eGFP	Sections/IF			2	59 ± 5.0
Controls	Sections/IF			14	0.0 ± 0.0

^*^Inter-lab comparison for AAV2-retro-CAG-tdTomato.

^**^Inter-lab comparison for AAV6-CMV-eGFP.

**Figure 4:**
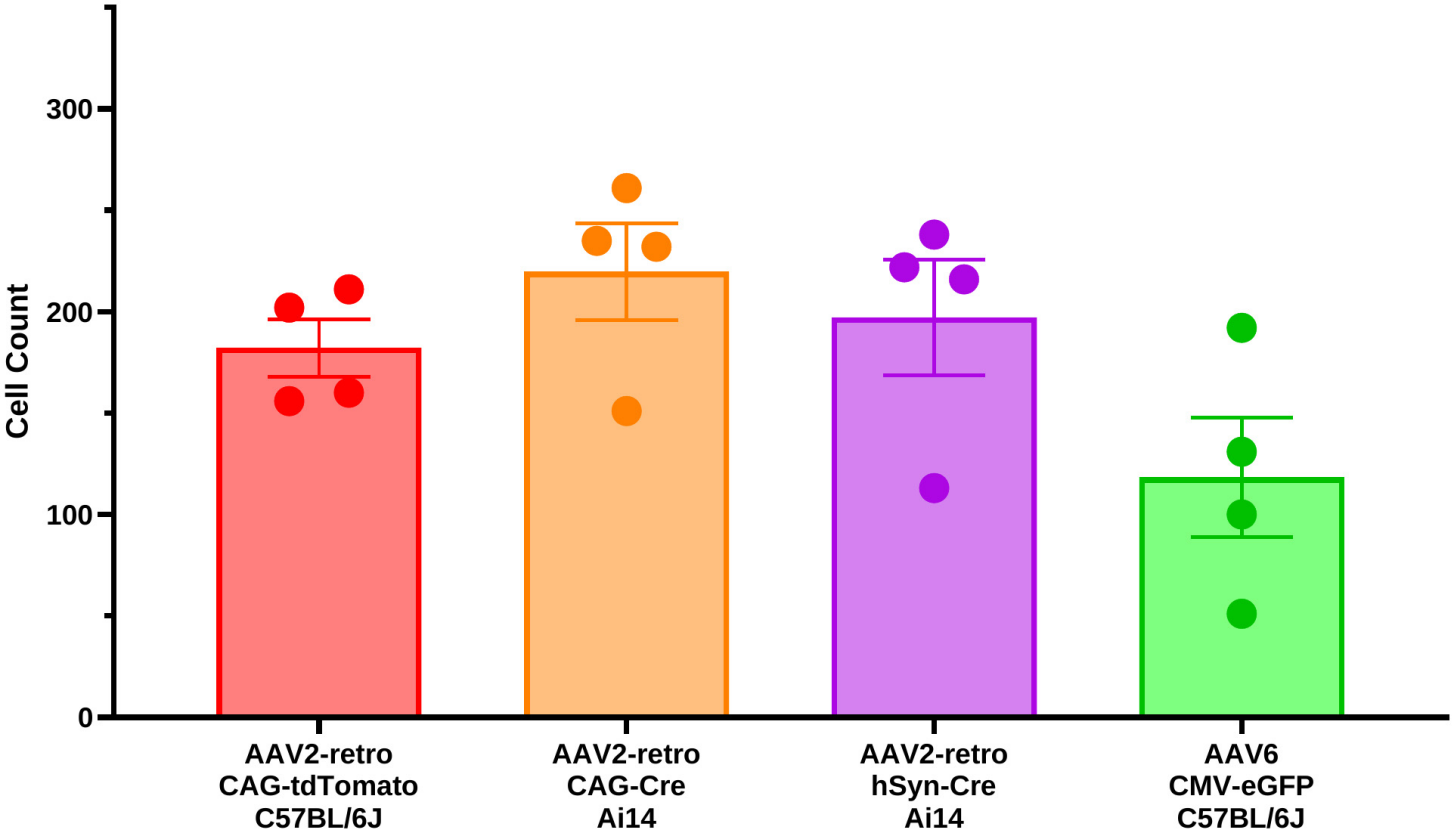
Quantification of labeled neurons in cleared whole trigeminal ganglia after intrastromal injection of four different AAV constructs in Laboratory A. AAV6-CMV-eGFP counts were quantified after GFP immunofluorescence amplification. Each point represents total detected cell count of one trigeminal ganglia per animal. No significant differences were detected among groups (*p* > 0.05, one-way ANOVA).

### Sectioned trigeminal ganglion analyses after intrastromal AAV injection

To further compare AAV-mediated labeling in sectioned tissue, TG sections were examined after intrastromal injection of AAV9-CAG-tdTomato, AAV-PHP.S-CAG-tdTomato, or AAV2-retro-CAG-tdTomato (Supplementary Figure 1) by Lab A. Consistent with the cleared whole-ganglion imaging, tdTomato-positive neurons were localized primarily within the dorsomedial region of V1. Quantification of tdTomato-positive neurons showed a significant effect of serotype on the number of labeled cells (Supplementary Figure 1A; one-way ANOVA, *p* < 0.01). *Post hoc* analysis revealed that AAV-PHP.S labeled significantly fewer neurons than either AV9 or AAV2-retro, whereas labeling after AAV9 and AAV2-retro was comparable. Notably, overall labeled cell counts in sectioned tissue were lower than those observed in cleared whole-ganglion preparations (Table 1). In Laboratory B, corneal neuronal labeling was evaluated after intrastromal injection of AAV1-CMV-mCherry, AAV2-7m8-CAG-eGFP, AAV6-CAG-eGFP, AAV8-CAG-eGFP, AAV9-CMV-eGFP and controls (Table 1). Very low efficiency was observed with AAV2-7m8-CAG-eGFP, a mid-level with AAV1-CMV-mCherry, AAV8-CAG-eGFP, AAV9-CMV-eGFP and a high level with AAV6-CAG-eGFP compared to controls. The high level of tropism of AAV6-CAG-eGFP derived from a combination of the promoter, with more neurons detected using the CAG than the CMV promoter, and the reporter gene, with more neurons detected using eGFP than mCherry even with antibody-enhanced detection, with the combination of the CMV promoter with mCherry resulting in very few labeled neurons (Figure 5G). AAV6-CMV-eGFP showed the lowest average labeling. One-way ANOVA revealed a significant difference between treatment groups (*p* < 0.0001), while *post-hoc* analysis identified significant differences between all comparison except AAV6-CAG-mCherry with AAV6-CMV-eGFP, and AAV6-CMV-eGFP with controls.

**Figure 5:**
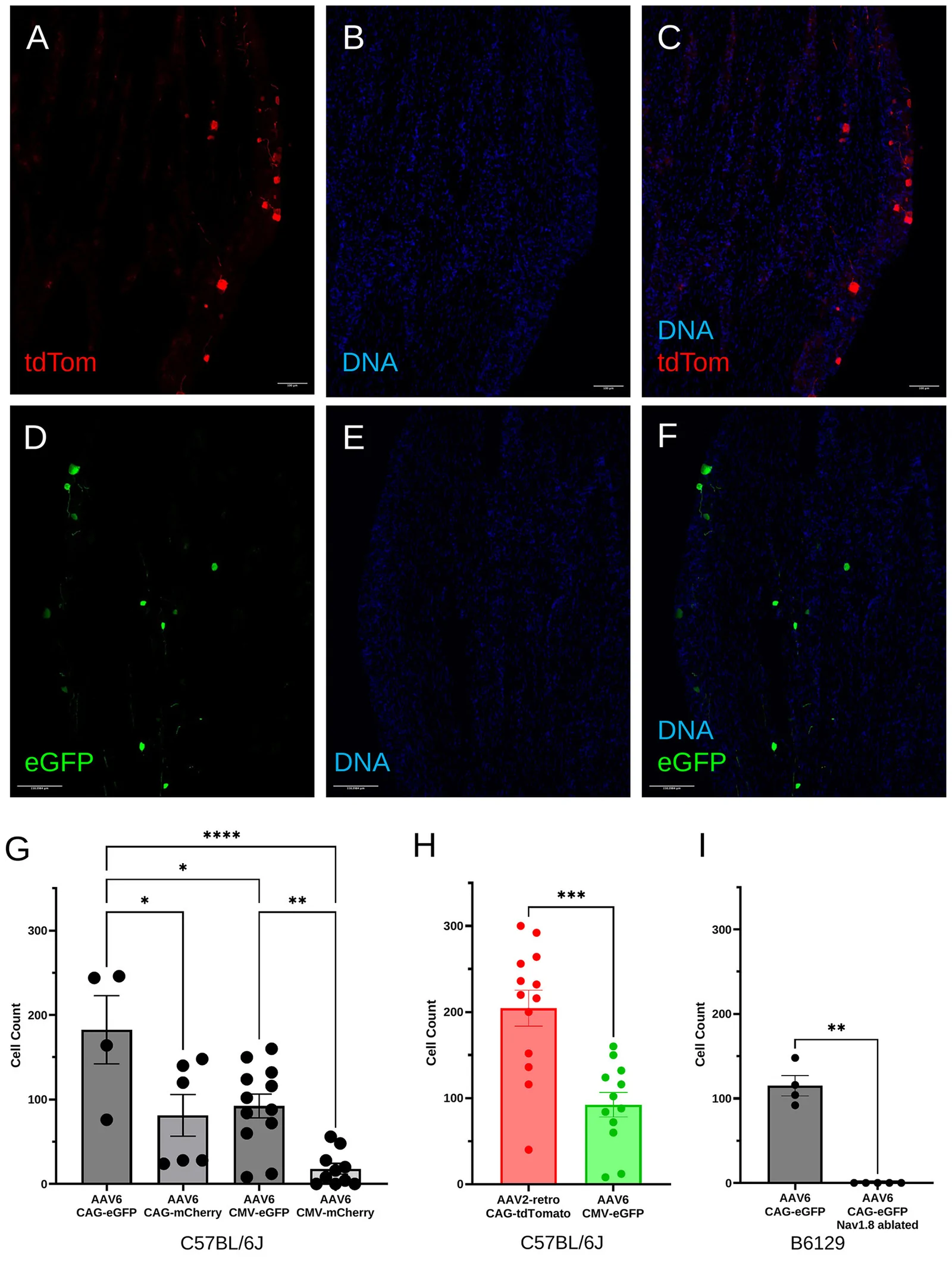
Corneal projecting neurons labeled by intrastromal injection of AAV2-retro-CMV-tdTomato or AAV6-CMV-eGFP in trigeminal ganglia sections, performed in Laboratory B. **(A)** Intrinsic tdTomato, **(B)** Hoechst 33342 and **(C)** merge from TGs injected with AAV2retro-CAG-tdTomato. **(D)** Antibody-enhanced eGFP, **(E)** Hoechst 33342 and **(F)** merge from TGs injected with AAV6-CMV-eGFP. 10× objective, 0.3 NA, Scale bar = 100 μm. Quantification of labeled neurons in trigeminal ganglia sections after intrastromal injection of five different AAV constructs. Each point represents the estimated total of labeled cell count in one trigeminal ganglion. **(G)** Comparison of AAV6 with CAG or CMV promoter and eGFP of mCherry reporter in C57Bl/6J mice. **(H)** Comparison of AAV2-retro-CAG-tdTomato and AAV6-CMV-eGFP in C57Bl/6J mice. **(I)** Comparison of AAV6-CMV-eGFP in wild type littermate and mice lacking nociceptor neurons (NaV1.8-ablated) in a B6129 genetic background. * *p* < 0.05, ** *p* < 0.01, *** *p* < 0.001, ****p < 0.0001, One-Way ANOVA or unpaired *t*-test.

Quantitation of the total number of labeled neurons after intrastromal injection of AAV2-retro-CAG-tdTomato and AAV6-CMV-eGFP was subsequently performed in parallel in two different laboratories to ascertain user-variability. In labeled TG sections performed in Laboratory B, the mean number of labeled neurons approximately double for AAV2-retro-CAG-tdTomato (*n* = 204 ± 21) compared to AAV6-CMV-eGFP (*n* = 92 ± 14) in 13 and 12 TG sections, respectively (Figures 5A–F, H, *p* < 0.01, paired *t*-test). Similar results were found for cleared TGs (*n* = 182 ± 14 AAV2-retro-CAG-tdTomato; *n* = 119 ± 30 AAV6-CMV-eGFP in 4 TGs each) performed in Laboratory A, confirming the greater degree of labeling with AAV2-retro-CAG-tdTomato (Figure 4). In mice lacking classic nociceptors via diphtheria-toxin mediated ablation of NaV1.8-expressing neurons, no AAV6-CAG-eGFP labeled neurons were detected (Figure 5I, *p* < 0.01, paired *t*-test).

### Consensus identification of corneal TG neurons by AAVs

To determine whether AAV-labeled TG neurons corresponded to cornea-projecting neurons identified by a conventional retrograde tracer, AAV-mediated labeling was compared with WGA tracing from the cornea. Intrastromal corneal injections of WGA were performed 4 weeks after AAV-retro-CAG-tdTomato, and animals were perfused 5-days later for whole ganglion clearing and light-sheet imaging (Figures 3A, B). Both tdTomato-positive and WGA-positive neuronal cell bodies were found in the same dorsomedial region of V1. Quantification of labeled neurons showed approximately 20% fewer AAV-positive neurons compared to WGA-positive neurons (Figure 3C). Despite this difference, there was substantial overlap between the two labeling approaches. Just over half of the total labeled population was double-labeled for tdTomato and WGA, whereas neurons labeled only by WGA represented nearly one-third of the total labeled population (Figure 3D). Dual labeling with FB tracing from the cornea and a 1:4 dilution of AAV2-retro-CAG-tdTomato was also validated (Supplementary Figure 2A). Quantification of labeled neurons showed ~50% fewer AAV-positive neurons compared to FB-positive neurons with the majority of tdTomato positive neurons also positive for FB (Supplementary Figure 2B). Because the vector was intentionally diluted for this experiment, the lower number of AAV-labeled neurons likely reflects the reduced dose administered. Undiluted AAV2-retro-CAG-tdTomato labeled approximately 4× the number of neurons when performed in Laboratory A and Laboratory B, detailed in previous section. These findings indicate that intrastromal AAV delivery labels a substantial proportion of WGA-identified and FB-identified cornea-projecting neurons with high concurrence, but that AAV-mediated labeling captures a somewhat smaller population compared with WGA tracing.

Potential differences in viral labeling associated with preferential transduction of specific subtypes of corneal neurons was further assessed by examining somatic sizes and co-expression of cornea-projecting neurons with CGRP. Across three serotypes, AAV9, PHP.S and AAV2-retro all with the CAG promoter, a subset representing approximately one-third of tdTomato-positive neurons was CGRP-positive (Supplementary Figures 1B, C). Independently, soma size was compared for AAV6-CMV-eGFP and AAV2-retro-hSyn-Cre labeled neurons (Figures 6A, B) in cleared trigeminal ganglia. Cell diameter measurements were performed in Napari by manually tracing the perimeter of each soma and converting the enclosed pixel area to an equivalent circular diameter in micrometers. Soma labeled by AAV6-CMV-GFP had a median equivalent diameter of 20.4 μm with two-thirds of soma between 15 μm and 25 μm and ranged from 10 μm to rare soma greater than 35 μm. Cross-lab validation showed consistent outcomes, although with smaller diameters, in sectioned tissue (Figures 6C, D). In TG sections, cell diameter analysis was performed in FIJI by measuring perpendicular lines spanning the soma. Soma labeled by AAV6-CMV-eGFP had a median diameter 16.9 μm with two-thirds of soma between 10 μm and 20 μm, ranging from 7–36 μm. A similar distribution was observed for AAV1-CMV-mCherry with two-thirds of soma between 10 μm and 20 μm, ranging from 7.8 μm−29.4 μm. Immune cells remained grossly quiescent, as assessed by the macrophage and dendritic cell markers CD206 (Supplementary Figure 3).

**Figure 6:**
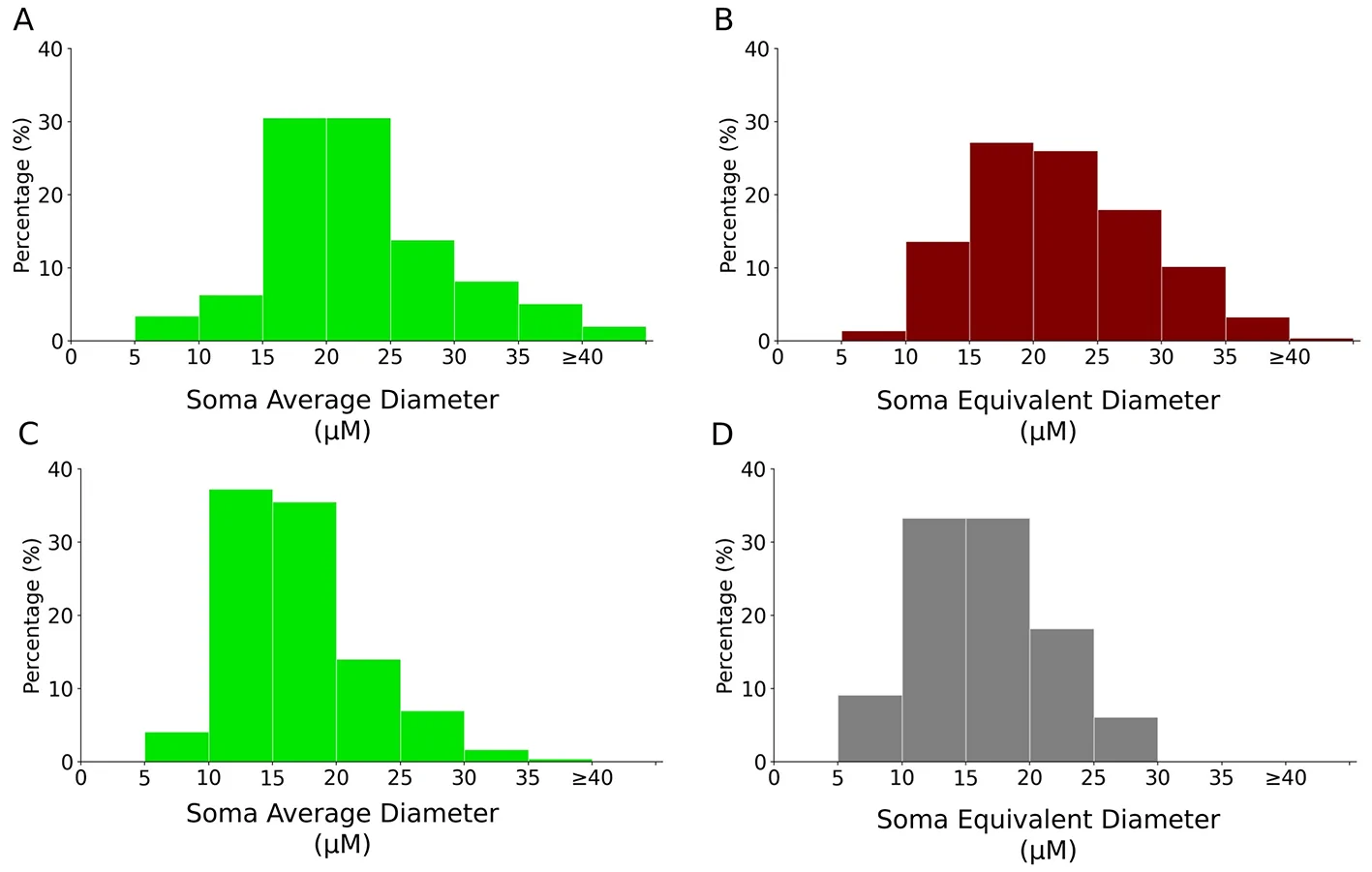
Size distribution of trigeminal ganglia cell soma labeled by injection of AAV into corneal stroma; 5 μM bins. **(A)** Equivalent diameter of soma labeled by AAV6-CMV-eGFP in cleared tissue. Diameter was determined by manually tracing the parameter of the cell soma and calculating the diameter of a circle with an area equal to that of the soma. **(B)** Equivalent diameter of soma labeled by AAV2-retro-hSyn-Cre in B6; Ai14 mice (tdTomato reporter) in cleared tissue. **(C)** Averaged diameter of soma labeled by AAV6-CMV-eGFP in sectioned tissue. Diameter was determined by taking two manual perpendicular measurements across the soma and averaging. **(D)** Averaged diameter of soma labeled by AAV1-CMV-mCherry in sectioned tissue.

### Corneal innervation is a strong indicator of AAV labeling efficacy

We next examined whether there was a correlation in the percentage of corneal nerve fibers expressing the viral reporter and the number of trigeminal ganglion neurons labeled. An ImageJ plugin, Corneal_Nerves, was developed to automate analyses of confocal images spanning the entire depth of a whole mount cornea. Reporter expression was observed in stromal, sub-basal plexus and inter-epithelial terminals from AAV6-CAG-eGFP transduced corneas. Regression analysis revealed a significant relationship between the innervation density in the sub-basal plexus (*R*^2^ = 0.72, *F*_(1,4)_ = 10.4, *p* = 0.03) whereas no significant relationship was observed for the stroma or intraepithelial terminals (Figure 7A). The extent of corneal labeling can be confounded by the relaxing cuts for flat-mounting, tissue artifacts and collateral damage during processing. To minimize these biasing factors, the degree of corneal reporter gene expression was also evaluated relative to total innervation using anti-b-III-tubulin co-labeling (Figures 7B–F).

**Figure 7:**
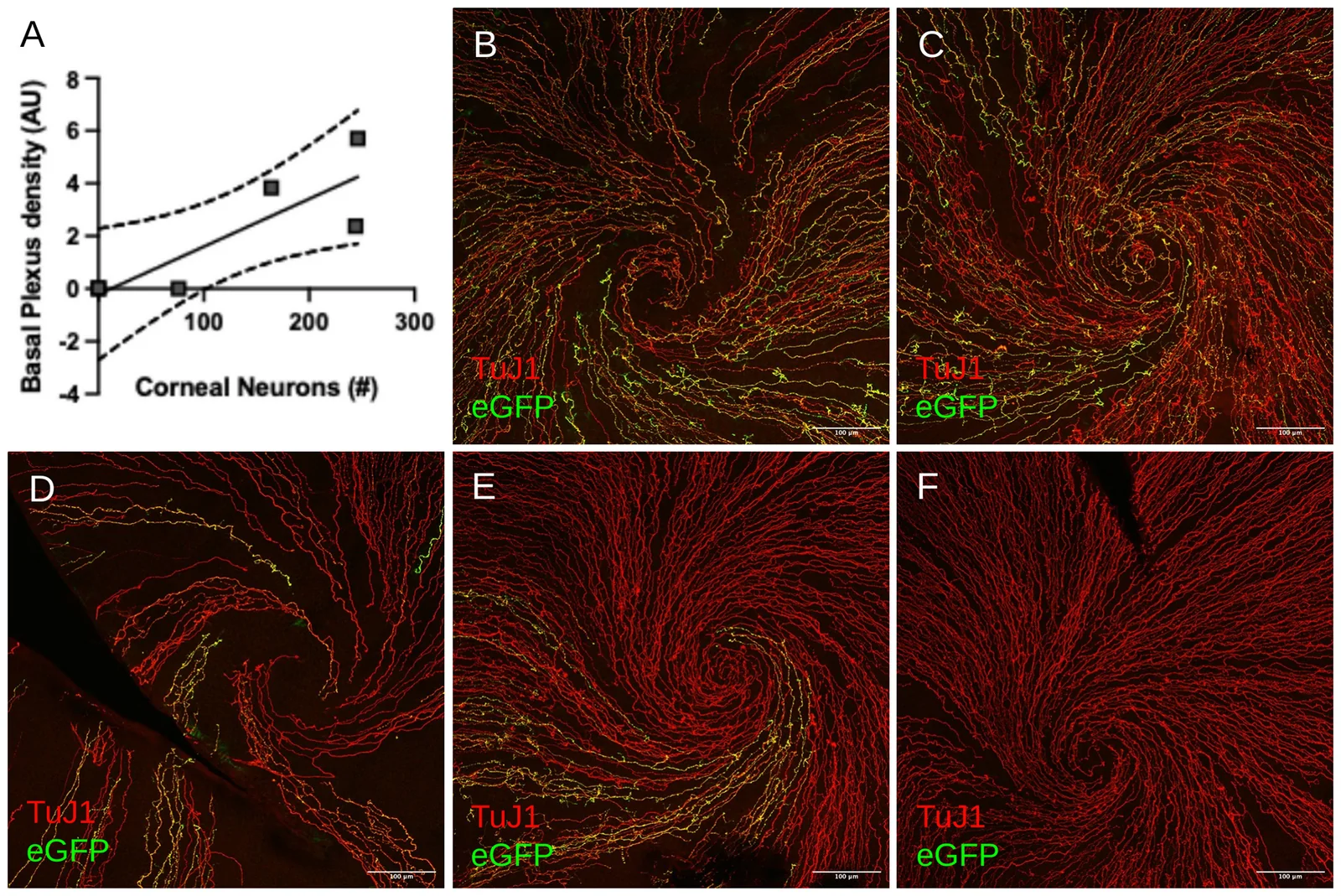
Corneas of stromal-injected mice were imaged for antibody-enhanced eGFP expression and β-III-Tubulin. Examples of central corneal whorls showing **(A)** 59%, **(B)** 40%, **(C)** 24%, **(D)** 12% and **(E)** control corneas co-labeling with anti-TuJ1 (magenta) and anti-GFP (green) are shown. Scale bars = 100 μm. **(F)** Regression analysis of the density of eGFP-labeled corneal sub-basal plexus nerve fibers with the number of labeled corneal neurons. *R*^2^ = 0.72, *p* = 0.03.

AAV-encoded tdTomato was detectable in peripheral corneal nerve terminals. Whole mount corneas from mice injected intrastromally with tdTomato-expressing AAV2-retro, AAV9, or AAV-PHP.S were imaged to assess tdTomato-labeled innervation of the corneal sub-basal nerve plexus (Supplementary Figures 4A, B). Automated analysis of confocal images revealed serotype-dependent differences in tdTomato-nerve density (Supplementary Figure 4A). One-way ANOVA showed a significant difference among groups, with AAV2-retro producing significantly greater tdTomato-labeled nerve density than either AAV9 or AAV-PHP.S, consistent with the relationship between the number of TGs transduced and the degree of corneal axon labeling. These findings indicate that AAV2-retro provides the most robust visualization of corneal afferent fibers among the compared serotypes, potentially reflecting a greater transduction efficiency and/or enhanced retrograde transport.

### Central projections after intrastromal AAV injection

Central processes of afferent neurons labeled by intrastromal injection of AAV2-retro-CAG-tdTomato and AAV9-CAG-tdTomato were assessed in 300 μm coronal sections spanning the trigeminal nucleus from subnucleus principalis (−4.7 mm bregma) to cervical spinal cord level C2. Confocal z-stacks were acquired at sites of tdTomato-labeled fiber termination (20×), with lower-magnification reference images collected for anatomical context (10×).

Labeled projections entered the brainstem via the ventromedial trigeminal sensory root, with the preponderance of fibers terminating ipsilaterally within the trigeminal brainstem nuclear complex and anterior cervical spinal cord. Between approximately −4.7 and −5.3 mm bregma, diffuse fibers extended dorsally from the ventral trigeminal sensory root into subnucleus principalis (Figure 8). The bulk of these fibers coursed near and parallel to the medial border of this region, lateral to the trigeminal motor nucleus, and terminated primarily in the dorsal extent of subnucleus principalis. In 2 of 16 animals, sparse projections from this population extended beyond the dorsal border of principalis into the extreme external-lateral parabrachial nucleus or Kölliker-Fuse nucleus (Figure 9).

**Figure 8:**
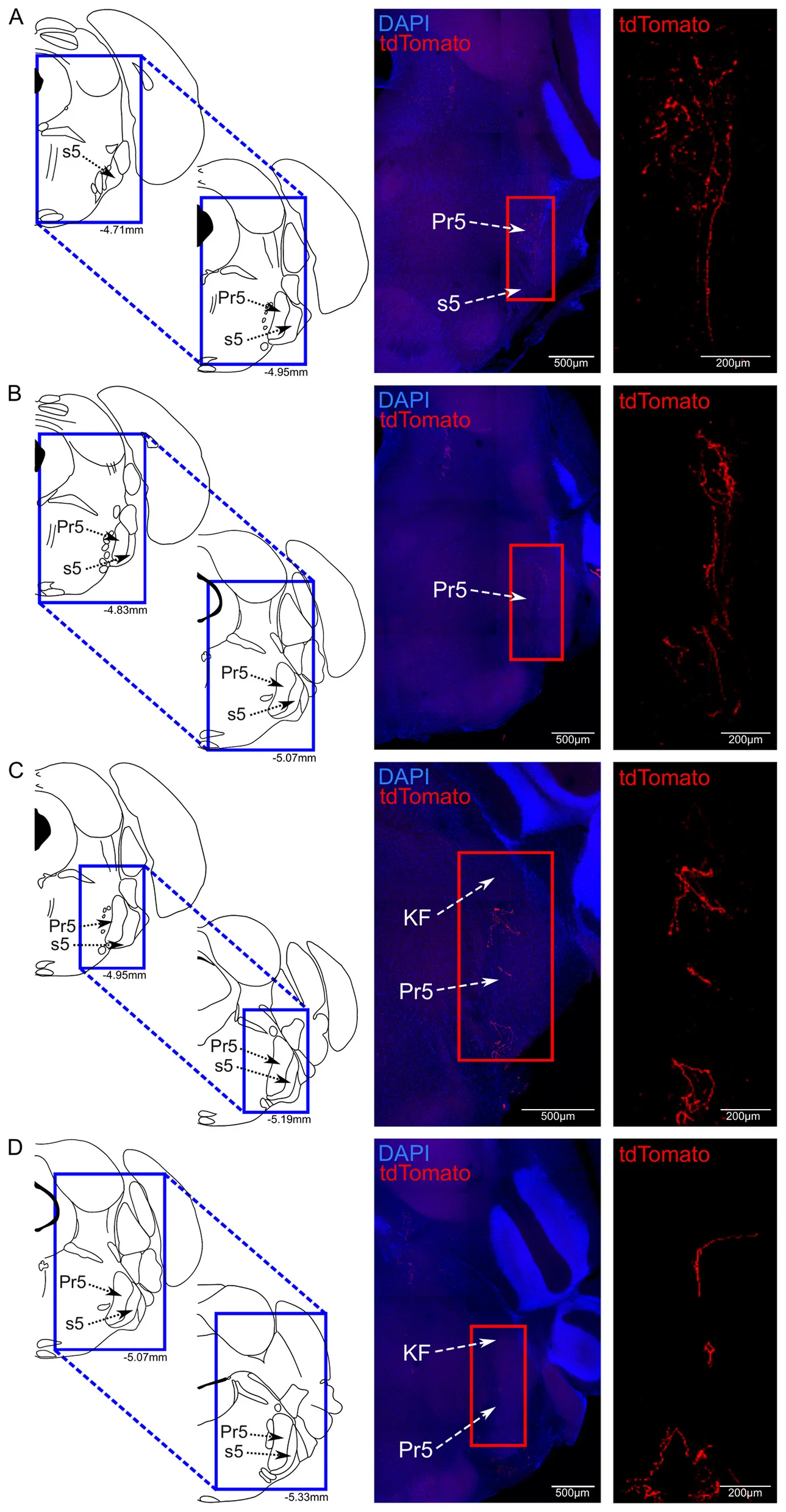
Projections to the subnucleus principalis of the trigeminal brainstem nuclear complex labeled by AAV2-retro-CAG-tdTomato (red) in cleared 300 μm thick sections stained with DAPI (blue). Images are maximum projections of z-stacks through each section. Diagrams at left demonstrate approximate anatomical locations and coronal planes at the beginning and end of the image stack. Detail images at far right correspond to the areas indicated by boxes in the overview images. **(A, B)** Labeled fibers originate from the ventral spinal trigeminal tract near the convergence of the sensory root. **(C, D)** Fibers primarily terminate at the dorsal extent of subnucleus principalis, inferior to the Kölliker-Fuse nucleus. Pr5, subnucleus principalis; s5, sensory root of the trigeminal nerve; KF, Kölliker-Fuse nucleus.

**Figure 9:**
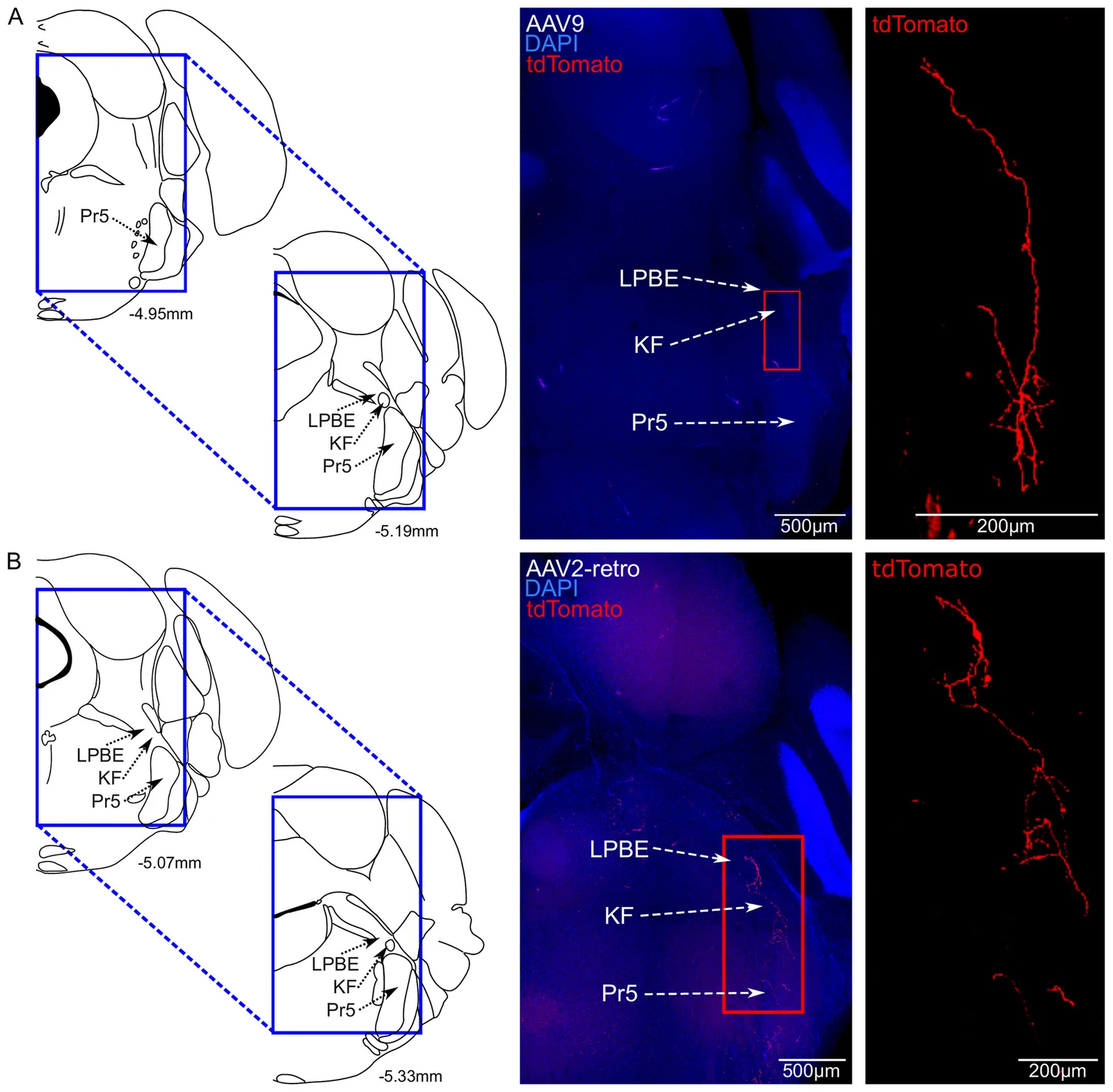
Sparse projections to the external-lateral area of the parabrachial nucleus labeled by **(A)** AAV9-CAG-tdTomato and **(B)** AAV2-retro-CAG-tdTomato (both red) in cleared 300 μm thick sections stained with DAPI (blue). Images are maximum projections of z-stacks through each section. Diagrams at left demonstrate approximate anatomical locations and coronal planes at the beginning and end of the image stack. Detail images at far right correspond to the areas indicated by boxes in the overview images. These fibers extend from the subnucleus principalis field, through the Kolliker-Fuse nucleus just medial to the lateral cerebral peduncle and terminate at the most lateral and ventral corner of the parabrachial nucleus. Pr5, subnucleus principalis; s5, sensory root of the trigeminal nerve; KF, Kölliker-Fuse nucleus; LPBE, lateral parabrachial nucleus external part.

From approximately −5.3 mm bregma onward, projections continued within the ventral trigeminal sensory root with no discernible terminations in subnucleus oralis or the rostral portion of subnucleus interpolaris. At ~-7.4 mm bregma (periobex level), a densely labeled terminal field spanned a rostrocaudal distance of approximately 0.3 mm, extending from the extreme caudal and ventral region of interpolaris into the most rostral, non-laminar portion of caudalis, anatomically consistent with the previously described Vi/Vc transition zone (Figure 10; Meng and Bereiter, 1996).

**Figure 10:**
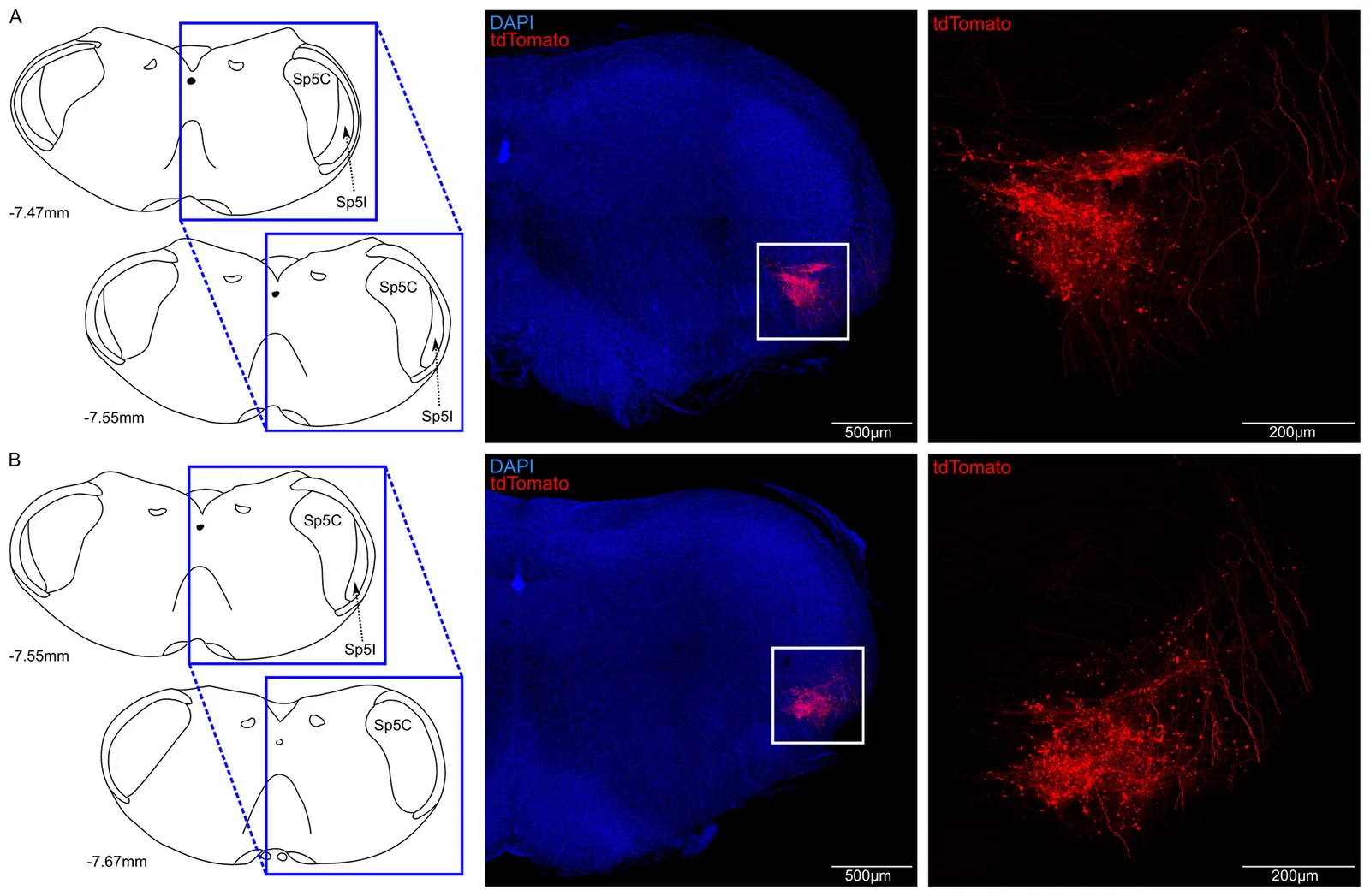
Projections to the subnucleus interpolaris to subnucleus caudalis transition zone (Vi/Vc) labeled by AAV2-retro-CAG-tdTomato (red) in cleared 300 μm thick sections stained with DAPI (blue). Images are maximum projections of z-stacks through each section. Diagrams at left demonstrate approximate anatomical locations and coronal planes at the beginning and end of the image stack. Detail images at far right correspond to the areas indicated by boxes in the overview images. **(A)** The dense and highly spatially localized terminal field beginning at the caudal and ventral region of interpolaris. **(B)** The field is restricted to the periobex level and continues ~0.3 mm in the rostracaudal axis into the rostral portion of caudalis. Sp5C, subnucleus caudalis; Sp5I, subnucleus interpolaris.

A third terminal field began at approximately −8.0 mm bregma (0.7 mm post-obex), where tract fibers spread into the dorsolateral trigeminal root and projected into the posterior portion of subnucleus caudalis, with fiber density increasing progressively in the caudal direction (Figure 11). Compared to the Vi/Vc field, this terminal field exhibited lower overall density but substantially greater spatial extent and was generally denser than the termination field within principalis. At the rostral aspect of this field, fibers appeared to terminate primarily in laminae I-II, with some additional fibers dispersed throughout deeper laminae. As the field transitioned into the dorsal horn, it shifted following the anatomical boundaries of the dorsal horn, becoming denser and more restricted to laminae I-II. Fiber density decreased at approximately the mid-C1 level, with the field ending entirely at approximately the rostral C2 level.

**Figure 11:**
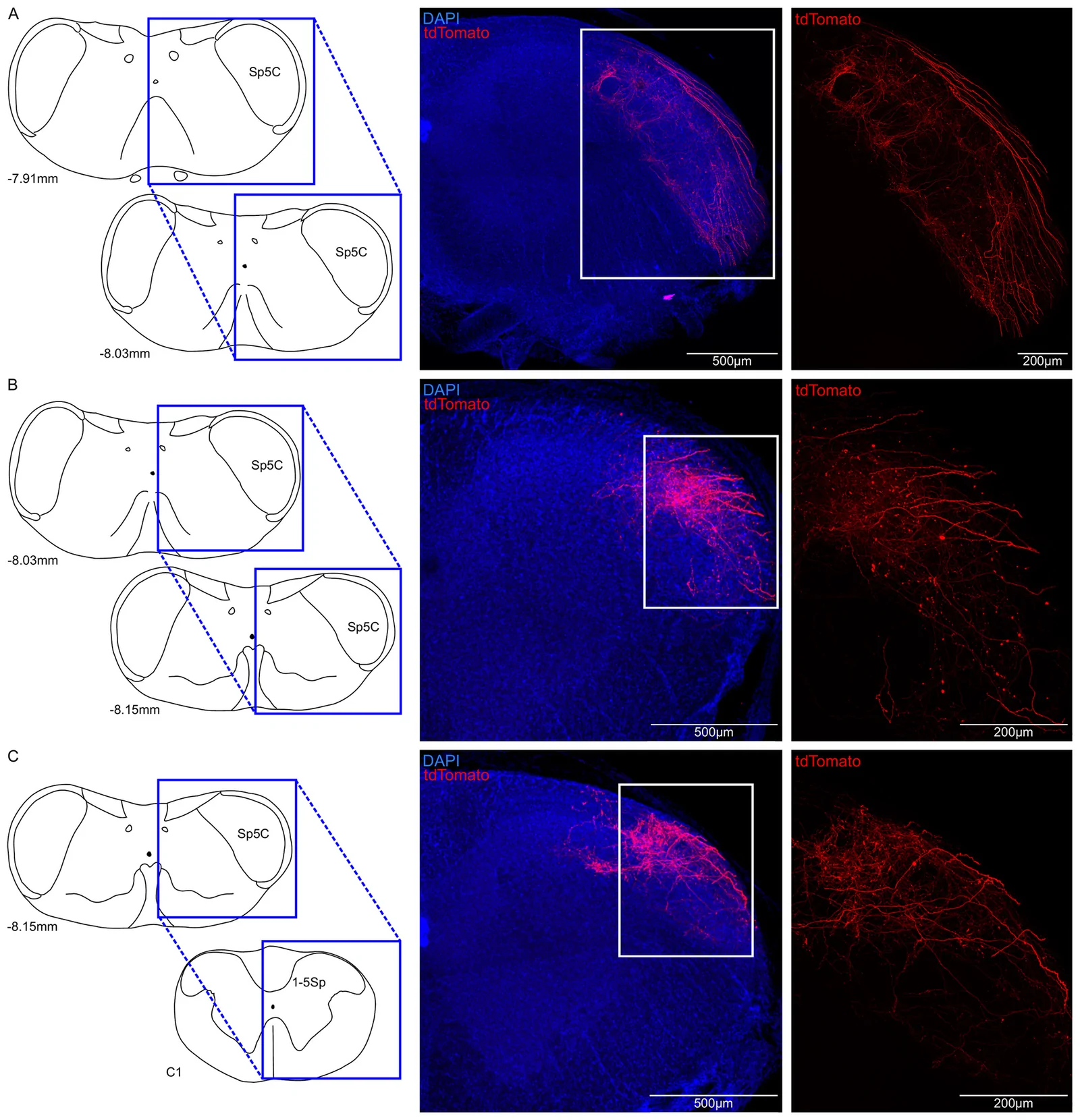
Projections to the subnucleus caudalis and cervical spinal cord transition zone (VcC1) labeled by AAV2-retro-CAG-tdTomato (red) in cleared 300 μm thick sections stained with DAPI (blue). Images are maximum projections of z-stacks through each section. Diagrams at left demonstrate approximate anatomical locations and coronal planes at the beginning and end of the image stack. Detail images at far right correspond to the areas indicated by boxes in the overview images. **(A)** Anterior areas of the terminal field are characterized by widely distributed fibers. **(B)** Posterior areas show increased density of fibers. **(C)** There is restriction to superficial laminae in the transition to spinal cord dorsal horn. Sp5C, subnucleus caudalis; 1-5Sp, laminae I-V of the spinal dorsal root.

## Discussion

The cornea is exquisitely tuned for pain due to the high density of nociceptive innervation (Muller et al., 2003; MacIver and Tanelian, 1993; Zander and Weddell, 1951; Rozsa and Beuerman, 1982; Muller et al., 1996, 1997). Despite the crucial role of this innervation in corneal homeostasis and its clinical relevance, experimental manipulations to probe selective functions have been constrained by a shortage of methods for modifying these neurons (Muller et al., 2003; Shaheen et al., 2014; Yang et al., 2018; Labetoulle et al., 2019; Feinberg et al., 2023; Liu et al., 2022; Jones and Marfurt, 1998; Okada et al., 2019). Prior to this study, AAV-mediated modification of corneal tissue was largely restricted to stromal and endothelial cells. Recent studies compared several AAV serotypes and delivery methods, concluding that AAV.rh10 was optimal (Cong et al., 2024, 2025). While the CMV promoter was only specified for their optimal serotype, it is likely its use contributed significantly to these findings. To expand the repertoire of labeling methods for corneal-projecting neurons, a panel of AAV serotypes, promoters, and reporters was systematically examined.

This screening identified a series of viruses demonstrating low, medium, and high labeling efficiency, each offering distinct advantages. Viruses yielding sparse labeling are ideal for anatomical tracing, high-fidelity 3D reconstruction, calcium imaging of individual neurons, and unambiguous somatotopic mapping of afferent terminals. Conversely, those producing dense labeling are optimal for comprehensive analyses querying population complexity and heterogeneity, such as degeneration/regeneration models or multi-omics applications (e.g., transcriptomics and proteomics).

Intrastromal injections were selected as the delivery route for all tested vectors. In a previous study, AAV1 delivered topically with or without corneal scarification was found to be ineffective at transducing corneal TG neurons (Dang et al., 2017). Properly performed and optimized, the injection limits epithelial damage to the needle entry site, delivers a precisely defined volume, and decreases the risk of the tracer labeling off-target structures by confining the bolus to a tissue compartment rather than permitting it to interact with the tear film. However, some spread of the injection toward the limbus or diffusion to other compartments cannot be anatomically excluded. Transgene expression in peri-corneal tissues was not assessed, and there remains the possibility that neurons innervating structures adjacent to the cornea may be labeled. Where strict anatomical specificity is required, injection volume and placement are the most likely determinants of peri-corneal spread. Injections in this study were placed centrally to maximize the distance from the limbus, and the smallest effective injection volume should be used to limit the risk of physical leakage. Promoter selection can help to restrict expression to neurons and limit expression in collaterally transduced keratocytes or other non-neuronal cells, but would not distinguish corneal afferents from those innervating adjacent tissue. Where that distinction is critical, direct examination of peri-corneal tissue for reporter expression should be made.

All viral serotypes, promoters and reporter genes selectively transduced neurons in the dorsomedial ophthalmic division of the TG with very few exceptions. Mirroring traditional tracing techniques, AAV constructs transduced a complement of neurons representative of native corneal innervation: the majority were classified as C-fibers and a smaller population as A-delta neurons, supported by a strong size-function relationship, co-expression of the neuropeptide CGRP and the loss of the smallest C-fiber neurons in the nociceptor-ablated mice. Co-labeling in an overlapping but not identical population with the classical retrograde tracers, WGA and FB (Felipe et al., 1999; Marfurt et al., 1989; Marfurt and Echtenkamp, 1988; Marfurt and Del Toro, 1987; LaVail et al., 1993; Nakamura et al., 2007; Hegarty et al., 2010; Aicher et al., 2013) further confirms the corneal identity of these neurons but raises the possibility of a preferential or selection bias despite otherwise equivalent-appearing populations. With the recent interest and advancements in the transcriptomes of these important neurons for ocular surface homeostasis, single cell transcriptomics of differentially labeled corneal projecting neurons may identify such biases.

Within- and across-laboratory analyses revealed that AAV2-retro-CAG exhibits higher efficiency than AAV6-CMV-eGFP, labeling the greatest number of corneal projecting neurons, while AAV6-CAG serves as a close second. When compared within the same serotype, the CAG promoter proved more effective than the CMV promoter, and eGFP proved a more reliable reporter than mCherry. Inter-laboratory variability, likely reflecting differences in post-injection expression time, tissue processing, and detection/quantitation methods, still yielded highly consistent relationships among the tested serotypes. Nevertheless, the experimenter's proficiency with intrastromal injections and post-harvest processing remains a critical variable. The most efficient vector constructs approached the numbers obtained with conventional retrograde tracers. In a direct comparison with WGA, AAV2-retro-CAG-tdTomato labeled approximately 20% fewer neurons. Detection threshold rather than transduction failure may account for a substantial fraction of the difference. Approximately 40% of eGFP-labeled neurons were detected only following antibody amplification, indicating that relying on virally expressed fluorescence may sometimes undercount transduced neurons.

Promoter selection is a critical decision in experimental design in the cornea. All promoters tested (CMV, CAG and hSyn) drive expression in corneal TG neurons. The hSyn promoter offers the potential advantage for intrastromal delivery as it is expected to restrict transgene expression selectively to neurons, whereas CAG and CMV are broad constitutive promoters that can support expression in neuronal and non-neuronal cells (Kugler et al., 2003; Nieuwenhuis et al., 2021). Non-neuronal expression of the reporter was observed in corneas injected with AAV6-CMV-mCherry but was not overly observed with other viruses. This distinction is particularly relevant in the cornea, where intrastromal AAV can enter and drive expression in stromal keratocytes and other local cells (Carlson et al., 2004; Mohan et al., 2011).

Fluorophore selection is another critical experimental consideration for spectral specificity and bleaching. For example, eGFP and mCherry support antibody-enhanced detection in both tissue sections and cleared whole-mount TGs and are compatible with many other fluorophores. Conversely, tdTomato endogenous fluorescence is easily detected, but its broad excitation and emission spectra can mask concurrent reporters. Notably, its spectra closely overlap with FM1-43, a traditional corneal projecting neuronal dye recently utilized to identify *Piezo2*-expressing mechanoreceptors (Villarino et al., 2023). tdTomato is highly resistant to bleaching, eGFP somewhat resistant and mCherry is readily bleached, with low bleachability beneficial for repeated, high-intensity imaging but detrimental for multiplex immunofluorescence imaging. As an alternative approach, a Cre-floxed permanent labeling system provides the distinct benefit of highly consistent reporter expression levels.

Methodologically, TG sections and cleared whole-mount tissues proved similarly effective for quantifying corneal-projecting TGs transduced by AAVs, and both formats benefit from antibody-enhanced eGFP and/or mCherry detection. While cleared, light-sheet imaged whole-mount samples trended toward a higher number of detected neurons, the difference was not statistically significant within the scope of this study.

Lastly, although sectioned and whole-mount trigeminal ganglia produced similar soma-size distributions, diameters measured in tissue sections were approximately 10 μm smaller. This difference may reflect, at least in part, differences in methodological approaches. In sectioned ganglia, perpendicular linear measurements were obtained from images of two-dimensional sections, which were not necessarily the neuron's maximal cross-section. Sections that do not pass through the maximal cross-sectional plane would yield smaller apparent diameters. By contrast, the whole-ganglion light-sheet z-stacks allowed each neuron to be followed through depth and the X-Y optical plane containing its largest soma profile to be selected before calculation of the area-equivalent circular diameter. The whole-mount approach would therefore be expected to produce larger estimates of soma diameter.

A primary advantage of AAV is the persistence of reporter expression following transduction. Corneal afferents maintained reporter expression for at least 3 months and, by comparison with transduced neurons in the retina or brain, may show robust expression for over a year without significant immunological complications (Kim et al., 2013; Muhuri et al., 2022). By contrast, traditional tracing methods frequently have narrow windows for optimal analysis due to the dynamics of retrograde transport. With retrograde transport, many traditional labeling methods see a loss of the tracer in the ocular surface corneal axons. The AAV6-CAG-eGFP reporter remained readily detectable for 3 months. Detection in corneal axons at the stromal, sub-basal plexus and intraepithelial layers required 2 weeks for initial antibody-enhanced detection. However, expression in soma was observable in the TG as early as 7 days post-transduction. Broad corneal axonal expression using other AAVs should be validated as AAV9-CAG-tdTomato was relatively weak and AAV1 reporter expression was observed to require more time to fill the axons. Time for expression, transduction efficiency, multiplicity of infection and capsid-promoter interactions may all influence outcomes (Bohlen et al., 2020).

This robust axonal expression allows researchers to evaluate labeling efficiency directly via the presence of the reporter gene in corneal nerve fibers, with specific AAV serotypes being more suitable for this type of analysis. This established correlation between labeling in the sub-basal plexus and the TG preserves valuable TG tissue, which can instead be reserved for specialized processing like spatial transcriptomics. To improve interpretability, co-labeling with anti-β-III-tubulin (a neuron-specific tubulin widely used to mark total corneal innervation) provides a reliable relative measure of axonal labeling that is less susceptible to artifactual loss of innervation or relieving cuts.

Axonal expression is not limited to peripheral corneal nerve fibers; AAV reporter expression was also observed in central projection areas for corneal afferents, including the parabrachial nucleus, the caudalis/interpolaris transition zone, and the subnucleus caudalis. These data support the utility of AAVs for the long-term expression of reporters or genetic tools designed to modify neuronal function from both peripheral and central axonal termini, as well as the somata.

The two most prominent caudal projection fields, at the Vi/Vc transition and in posterior Vc extending into the C1–C2 dorsal horn, closely match the principal central representations of corneal input identified by conventional tracing, corneal stimulation-induced c-Fos protein, and electrophysiological recordings in rats (Marfurt and Del Toro, 1987; Meng et al., 1997; Hirata et al., 2004; Meng and Bereiter, 1996). These regions appear to support partly distinct aspects of corneal sensation. Corneal-responsive neurons in the Vc/C1 transition region have receptive-field and response properties resembling nociceptive neurons in the medullary and spinal dorsal horn and contribute to processing corneal pain (Meng and Bereiter, 1996; Hirata et al., 2000). In contrast, neurons near the Vi/Vc transition are strongly influenced by ocular surface cooling and drying and participate in reflex tearing and blinking, consistent with a role in ocular surface homeostasis (Meng and Kurose, 2013; Hirata et al., 2004; Kurose and Meng, 2013). The diffuse projection observed in dorsal subnucleus principalis is noteworthy. A rostral projection to principalis was reported after corneal tracing in primates (Marfurt and Echtenkamp, 1988), whereas rat studies have found corneal labeling restricted to caudal trigeminocervical regions (Marfurt and Del Toro, 1987; Panneton et al., 2010). The difference in the present mouse study could reflect species differences or greater sensitivity of AAV reporter expression. Sparse labeled fibers extending toward the extreme lateral parabrachial or Kölliker-Fuse region were observed in 2 of 16 animals. This projection would be consistent with previous studies indicating a direct monosynaptic input from the TG to the lateral parabrachial nucleus, a pathway implicated in the affective and motivational dimensions of craniofacial pain (Rodriguez et al., 2017).

Taken together, this validated toolbox of AAV viruses offers broad applicability for anatomical studies mapping central to peripheral projections, functional studies utilizing optogenetic or chemogenetic manipulations for activity monitoring, and targeted gene therapy in corneal projecting neurons. This survey of AAV serotypes may show similar efficacy for other peripheral sensory innervated targets as the corneal niche is unique in its constellation of neurons tuned for highly sensitive nociceptive function but has the same types of neurons as elsewhere in the trigeminal and dorsal root ganglia. Tested for reproducibility and rigor across independent laboratories, these findings provide clear, structured guidance on selection criteria for future experimental designs.

## Data Availability

The original contributions presented in the study are included in the article/supplementary material, further inquiries can be directed to the corresponding authors.
