# Supplementary material for: Adeno-associated virus gene delivery targeting corneal-projecting trigeminal ganglion neurons
Source: Front Cell Neurosci. 2026 Oct 22;20:1925806. doi: 10.3389/fncel.2026.1925806 (PMC13735549; doi:10.3389/fncel.2026.1925806)
Supplement: Supplementary file 1 [file Data_Sheet_1.pdf]

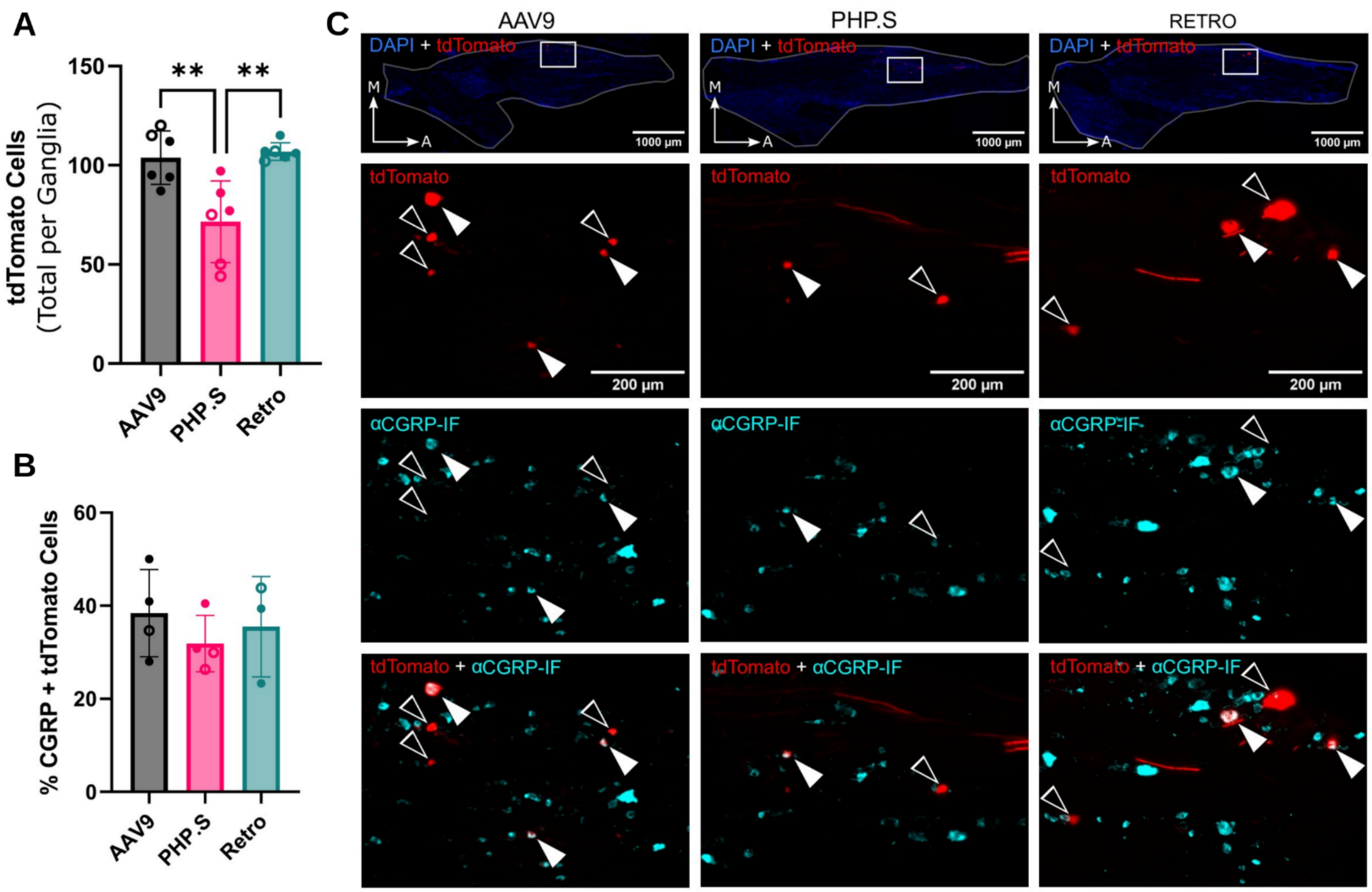

Supplemental Figure 1. Trigeminal ganglia labeled neurons after intrastromal corneal injections of three different serotypes (AAV9, AAV-PHP.S and AAV-retro) in sectioned tissue. **(A)** A greater number of tdTomato-positive neurons was observed after AAV9 and AAV-retro injections compared to PHP.S. (1-Way ANOVA, $p<0.01$). Open circles, female mice; solid circles, male mice. ** $p<0.01$. **(B)** Immunohistochemical analysis on a subset of samples revealed comparable proportions of CGRP-positive neurons among tdTomato-labeled cells across serotypes. **(C)** Representative trigeminal ganglia cryosections with cells labeled by each serotype. Top panels: overview with DAPI. Lower panels: details of tdTomato, αCGRP-IF, and co-labeling in areas indicated by box in overview image. Open arrows indicate tdTomato cells with no αCGRP-IF labeling. Solid arrows indicate tdTomato cells co-labeled with αCGRP-IF.

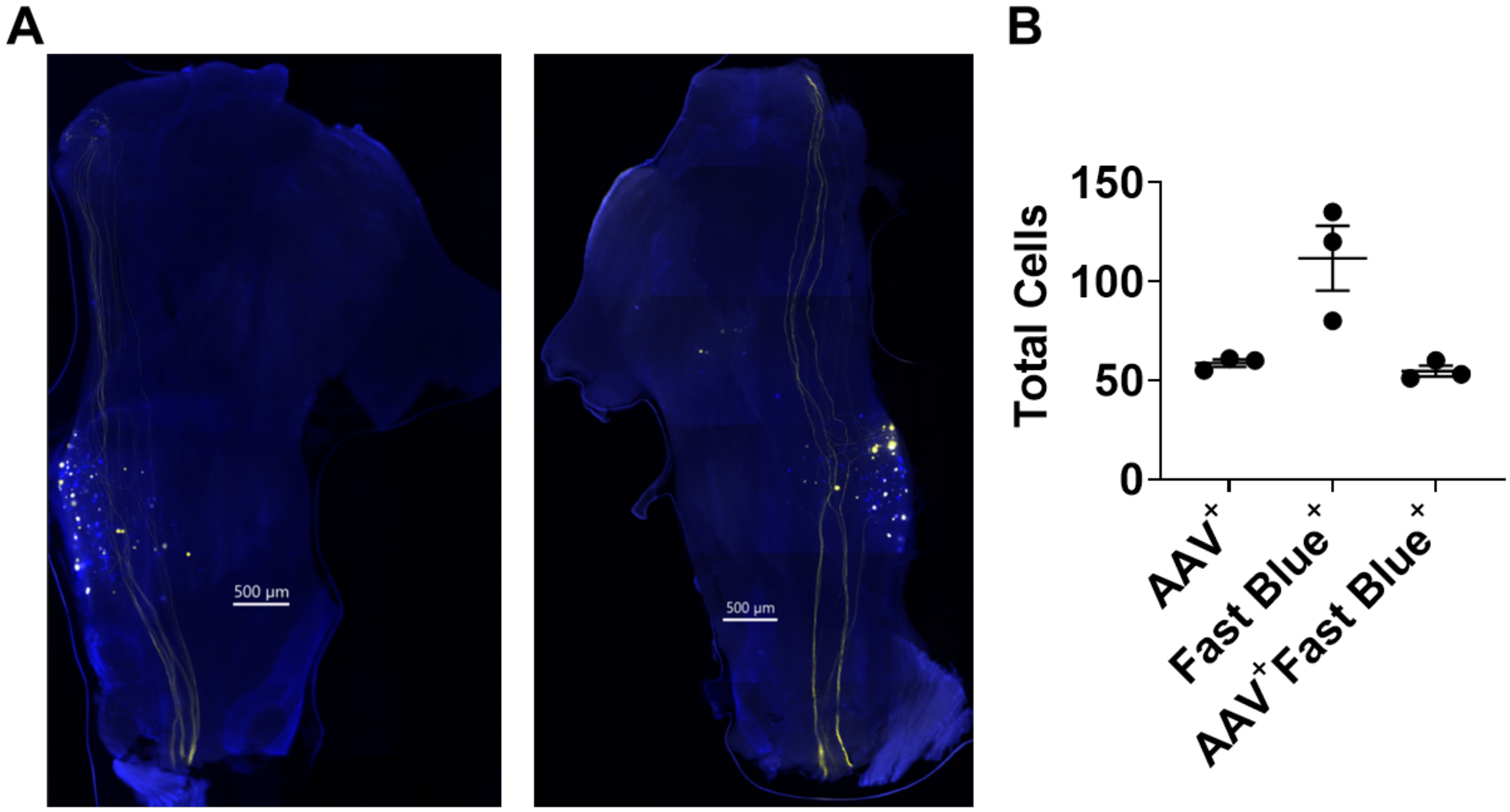

Supplemental Figure 2. Comparison of AAV2-retro and Fast Blue labeling of corneal afferents. **(A)** Representative whole mounts of trigeminal ganglia following intrastromal injection of AAV2-retro-CAG-tdTomato (1:4 dilution, 1-2 µL) and retrograde tracing with Fast Blue. AAV was injected 4 weeks prior to tissue collection. Fast Blue was injected 3 days prior to tissue collection. Trigeminal ganglia were processed and imaged as detailed in Yun et al., 2025. **(B)** Quantification of total labeled cell bodies positive for AAV (tdTomato), Fast Blue, or both, showing a greater number of Fast Blue labeled cells relative to AAV-labeled or double-labeled cells.

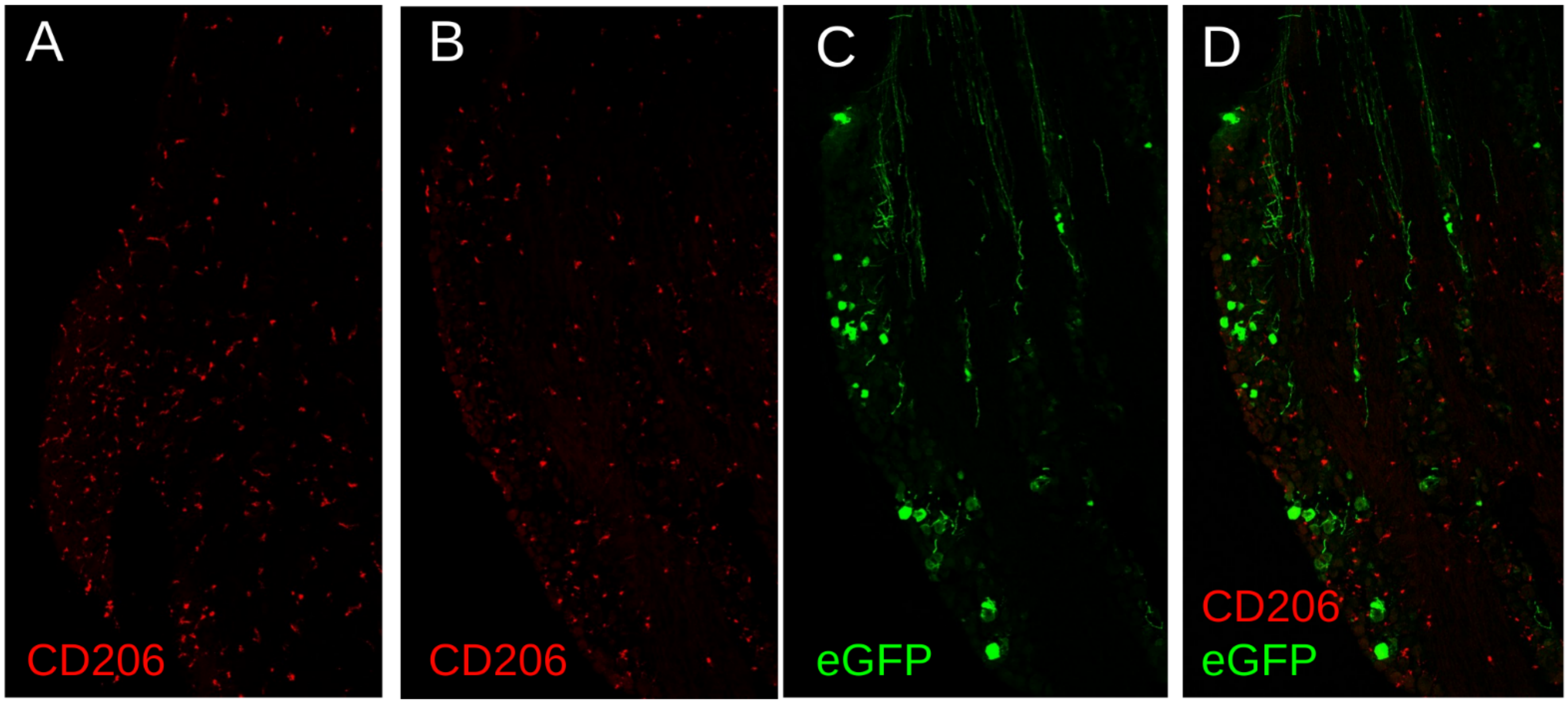

Supplemental Figure 3. Immune cells remained grossly unaffected following AAV6-CAG-eGFP administration. **(A)** Control TGs have a broad distribution of CD206-positive cells. **(B, C, D)** A similar distribution of CD206-positive cells was observed in TG transduced by AAV6-CAG-eGFP. **(B)** anti-CD206-IF, **(C)** anti-eGFP for the viral reporter gene and **(D)** merged image. Scale bar = 100μm.

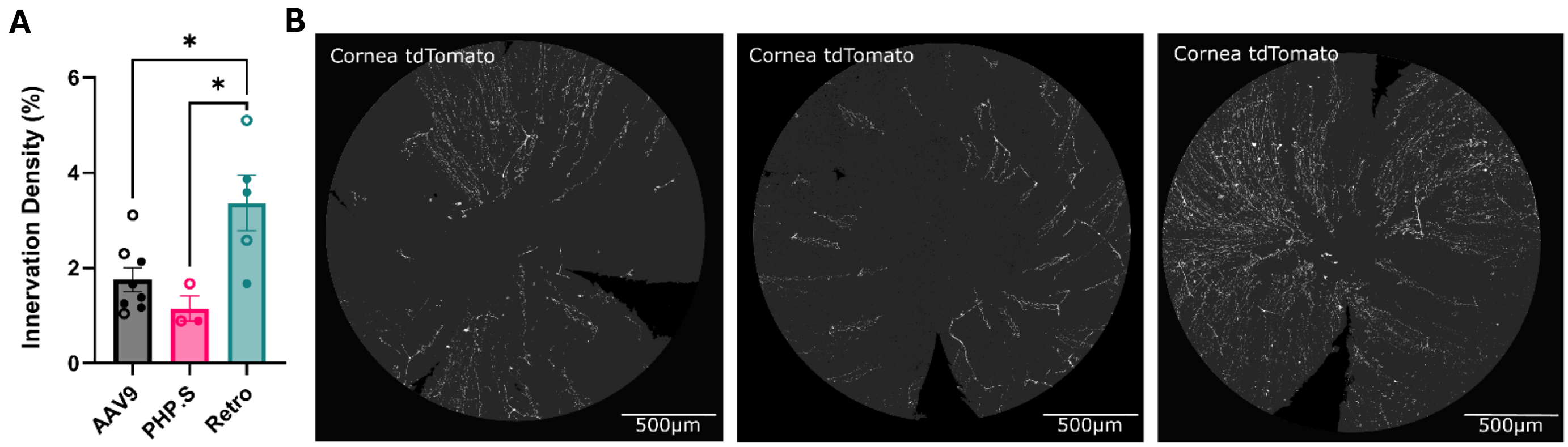

Supplemental Figure 4. Corneas of stromal-injected mice were imaged for tdTomato expression. **(A)** Automated analysis of the corneal sub-basal plexus revealed serotype-dependent differences in tdTomato-labeled nerve density. **(B)** In a 2000 µm central region of the cornea, AAV2-Retro injected mice showed significantly higher nerve area coverage compared to AAV9 and AAV-PHP.S (1-Way ANOVA, * $p<0.05$).
